# Complex and variable regulation of ΔNp63 and TAp63 by TGFβ has implications for the dynamics of squamous cell epithelial to mesenchymal transition

**DOI:** 10.1038/s41598-024-57895-1

**Published:** 2024-03-27

**Authors:** Zuzana Pokorná, Zuzana Tylichová, Borivoj Vojtesek, Philip J. Coates

**Affiliations:** 1 Research Center of Applied Molecular Oncology (RECAMO), Masaryk Memorial Cancer Institute, Zluty Kopec 7, 656 53 Brno, Czech Republic; 2 Present Address: State Institute for Drug Control, Šrobárova 48, 100 41 Praha 10, Czech Republic

**Keywords:** ΔNp63, TAp63, Squamous cell carcinoma, TGFβ, EMT, Head and neck cancer, Oral cancer, Cancer

## Abstract

TGFβ has roles in inflammation, wound healing, epithelial to mesenchymal transition (EMT), and cancer stem cell states, and acts as a tumor suppressor gene for squamous cell carcinoma (SCC). SCCs are also characterized by high levels of ΔNp63, which induces epithelial cell phenotypes and maintains squamous stem cells. Previous studies indicate a complex interplay between ΔNp63 and TGFβ signaling, with contradictory effects reported. We investigated the effects of TGFβ on p63 isoform proteins and mRNAs in non-malignant squamous and SCC cells, and the role of either canonical or non-canonical TGFβ signaling pathways. TGFβ selectively increased ΔNp63 protein levels in non-malignant keratinocytes in association with SMAD3 activation and was prevented by TGFβ receptor inhibition, indicating activation of canonical TGFβ pathway signaling. *TP63* isoform mRNAs showed discordance from protein levels, with an initial increase in both *TAP63* and *ΔNP63* mRNAs followed by a decrease at later times. These data demonstrate complex and heterogeneous effects of TGFβ in squamous cells that depend on the extent of canonical TGFβ pathway aberrations. The interplay between TGFβ and p63 is likely to influence the magnitude of EMT states in SCC, with clinical implications for tumor progression and response to therapy.

## Introduction

The transforming growth factor-β (TGFβ) family is comprised of TGFβ1, 2, and 3, bone morphogenic proteins (BMPs), nodal, and activin. Within the family, TGFβ is a multi-functional cytokine that regulates proliferation, differentiation, and migration, and plays roles in the resolution of inflammation, fibrosis, wound healing, and cancer (reviewed in^1–3^). TGFβ acts through its membrane receptor (TGFBR1/TGFBR2) that phosphorylates SMAD2/3 to transcriptionally regulate target genes. In addition to canonical SMAD-dependent signaling, SMAD-independent (non-canonical) effects may occur through ERK, MAPK, PI3K/Akt, or Rho pathways, and nearly all adult cell types are responsive to TGFβ in a context- and concentration-dependent manner^2,3^. For example, although TGFβ is generally considered an anti-proliferative cytokine for epithelial cells, it may either inhibit or promote the proliferation of endothelium and some mesenchymal cells, depending on context and concentration^4^. TGFβ is normally a tumor-suppressive and anti-proliferative cytokine for epithelial tissues, and tumors therefore often show aberrant TGFβ signaling due to inactivating mutations in SMADs or TGFBR, or in genes that are downstream of TGFβ^2,3,5^. In particular, alterations in SMADs, especially but not only *SMAD4*, and in *TGFBR2,* are recurrently found in some tumor types in association with decreased TGFβ signaling. Moreover, a variety of tumorigenic events prevent TGFβ signaling to mediate growth arrest in cancer cells, including loss of CDKN1A activation through Akt-mediated FOXO regulation, or Miz1 impairment of Myc activity impairing CDKN2B expression, resulting in a loss of the cytostatic effect of TGFβ signaling in tumor cells (reviewed in^2,3,6^). Thus, these alterations remove at least some of the tumor-suppressive effects of TGFβ while retaining other tumor-promoting effects that include suppressing anti-tumor immune responses and inducing the production of pro-mitogenic factors in the tumor microenvironment^2,3,6^. One of the most notable properties of TGFβ is to stimulate epithelial-to-mesenchymal transition (EMT), which is pro-migratory and induces a cancer stem cell (CSC) phenotype^5^. In human cancer, TGFβ signaling inhibition is common in squamous cell carcinomas (SCC)^6^, acting as an oncogenic driver^7^, and the level of inhibition has been shown to associate with tumor progression^8^.

The *TP63* gene is a member of the *TP53* family of transcription factors encoding two major protein variants, TAp63 and ΔNp63, with opposing or independent effects^9–11^. In particular, TAp63 contains a p53-like transactivation domain and has tumor-suppressor effects, whereas ΔNp63 lacks this domain and has oncogenic activities^11,12^. TAp63 and ΔNp63 are expressed in distinct cell types in normal tissues and are independently regulated to achieve their functions^9–11^. ΔNp63 is present in basal and parabasal cells of normal squamous epithelium, is a key inducer of squamous cell lineage commitment, and acts to maintain the undifferentiated squamous cell phenotype, cell survival and proliferation^9–11,13,14^. Overexpression of ΔNp63 is a characteristic feature of SCC^9,10,15–18^, and is associated with poor prognosis and therapy resistance^19–23^. TAp63 may antagonize ΔNp63 in squamous tissues to reduce proliferation, and promote rather than inhibit cell differentiation. TAp63 is present in embryonic squamous epithelium but not in adult squamous cells, although some SCCs show low levels of TAp63 in association with improved survival in keeping with roles in growth arrest, apoptosis and differentiation^9,12^.

Previous reports indicate complex cross-talk between p63 and TGFβ, with conflicting results. For example, p63 inhibition was reported to reverse EMT and TGFβ-dependent cell proliferation and migration^24–28^, and to downregulate *Tgfb2* and *Tgfb3*^29^. Similarly, ΔNp63 induced TGFBR2^30^ and enhanced SMAD2 activity^31^, in keeping with a requirement for ΔNp63 in TGFβ-induced EMT^32^. In contrast, ΔNp63 has also been reported to inhibit TGFβ signaling by repressing TGFβ1/2 and TGFBR^33^, and p63 inhibition enhanced TGFβ-induced metastasis^34^. TGFβ may also regulate p63, again with conflicting data: TGFβ enhanced p63 activity without changing its level^35^, or increased ΔNp63 protein/mRNA levels^28,35–37^. On the contrary, TGFβ was reported to decrease^26,38^, and inhibition of SMAD activity to increase ΔNp63^38,39^. In vivo, *Tgfb1* deletion in basal squamous cells caused a dose-dependent reduction of p63 in these cells at birth, with a subsequent increase in suprabasal cells in hemizygous *Tgfb1*^+/-^ mice, revealing cell-type and dose- and time-dependent effects of TGFβ on p63 in normal keratinocytes^40^.

These data indicate that p63 and TGFβ have both complementary and opposing functions, in which each promotes the CSC state but TGFβ induces EMT whereas ΔNp63 maintains an epithelial cell phenotype^3,5,10,11,13,17,18^. In view of the important effects of TGFβ and ΔNp63 in SCC development and progression, and the discrepant results reported for their regulatory connections in normal or cancer cells of different tissue origins, we performed an in-depth analysis of non-malignant and malignant squamous cells using consistent growth conditions and exposures to clarify the effects of TGFβ on p63 in this cell type. We also examined TAp63 and ΔNp63 isoforms and studied whether the effects of TGFβ on p63 are associated with SMAD activation. The experiments reveal a complex response system that involves cell-dependent biphasic induction/repression of p63 mRNA and protein. The variable responses we uncover have implications for the role of TGFβ on the magnitude of EMT states in SCC, with clinical implications for tumor progression, metastasis and response to therapy.

## Results

### TGFβ induces variable concentration-dependent increases in ΔNp63 protein

**Figure 1:**
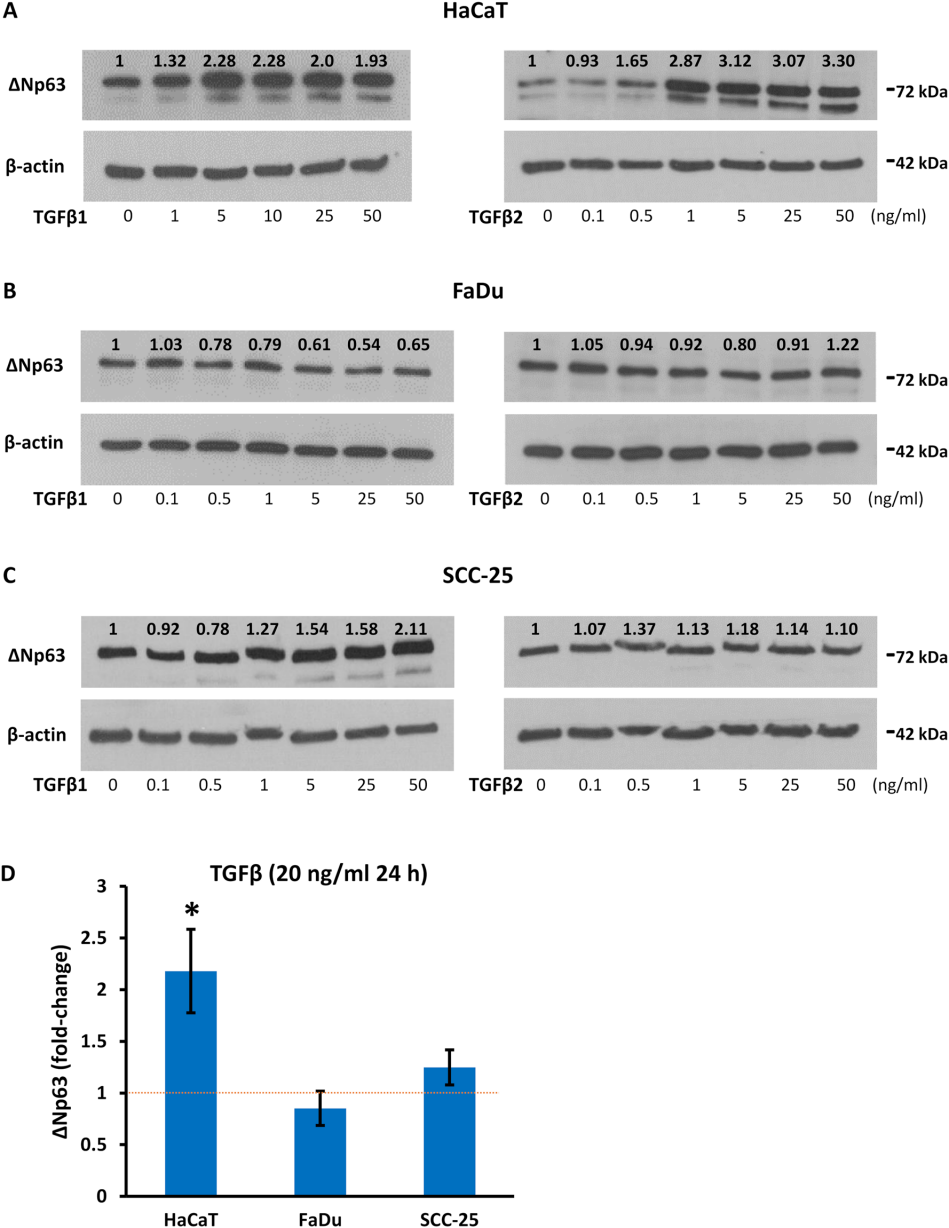
Concentration- and cell-dependent increase in ΔNp63 protein levels after TGFβ treatment. (**A**–**C**) Representative Western blots of ΔNp63 in (**A**) HaCaT, (**B**) FaDu, and (**C**) SCC-25 cells treated with increasing concentrations (from 0 to 50 ng/ml) of TGFβ1 (left) or TGFβ2 (right) for 24 h. β-actin was used as the loading control. Numbers above each lane show the relative level of ΔNp63 measured by densitometry and normalized to β-actin in the same sample. Control cells cultured in the absence of TGFβ are designated as 1.0. (**D**) Average fold-change in ΔNp63 protein in each cell line after treatment with 20 ng/ml TGFβ for 24 h compared to untreated control cells and normalized to β-actin (mean ± SEM; n = 3—5 biological replicates). *p < 0.05 compared to control cells. The dotted red line represents the average level in untreated cells.

We examined the effects of TGFβ in non-tumorigenic HaCaT keratinocytes that retain full squamous cell differentiation capacity, and in FaDu and SCC-25 malignant SCC cells that represent the atypical and mesenchymal molecular subtypes of SCC, respectively^41^. During these experiments, cells were plated at initial densities to achieve similar confluency at the end of the experiment, and we were careful to ensure that cells never reached confluency, which may itself have influenced p63 isoform levels. Cells were initially treated for 24 h with increasing concentrations of TGFβ1 or TGFβ2, and assessed by Western blotting for ΔNp63 (the predominant isoform in these cells^42^). TGFβ1 and TGFβ2 upregulated ΔNp63 protein levels in HaCaT cells at 1 ng/ml and higher concentrations, plateauing above 5 ng/ml (Fig. 1A). FaDu cells showed no increase in ΔNp63 after treatment with either TGFβ1 or TGFβ2 at any concentration (Fig. 1B) and SCC-25 cells showed a lesser and more variable response than HaCaT, with higher concentrations required to achieve induction (Fig. 1C). From these data, further replicate experiments were performed using 20 ng/ml TGFβ1 or TGFβ2 for 24 h, revealing a similar magnitude of response to both TGFβ forms, consistent with previous data on the equivalent potency of TGFβ1 and TGFβ2 in epithelial cells^43,44^. Pooling the replicate data showed an average 1.94-fold increase of ΔNp63 in HaCaT cells only (p < 0.05) (Fig. 1D). Thus, TGFβ induces ΔNp63 protein in non-tumorigenic HaCaT keratinocytes, and has variable and minimal effects in malignant SCC cells.

### ΔNP63 mRNA is inversely correlated with ΔNp63 protein in HaCaT cells after TGFβ treatment

**Figure 2:**
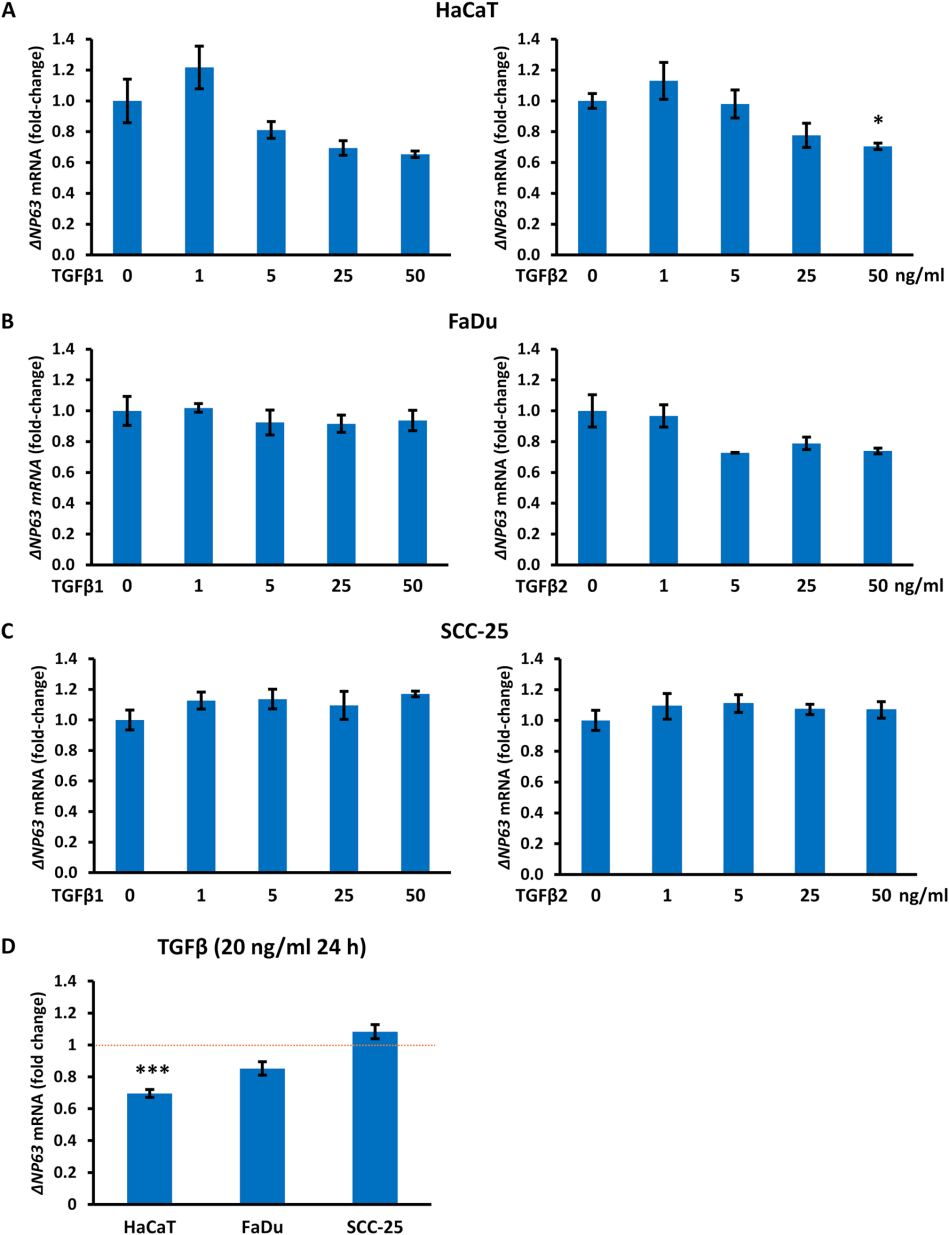
Concentration- and cell-dependent decrease in *ΔNP63* mRNA after TGFβ treatment. (**A**–**C**) The indicated cell lines were treated with 0–50 ng/ml of TGFβ1 (left) or TGFβ2 (right) for 24 h. RT-qPCR was performed for *ΔNP63* and *ACTB* (β-actin) for normalization. The bar charts indicate relative changes in *ΔNP63* mRNA compared to cells cultured in the absence of TGFβ. Error bars represent SEM (n = 3 biological replicates). (**D**) Average fold-change in *ΔNP63* mRNA in each cell line after incubation with 20 ng/ml TGFβ for 24 h compared to untreated cells (mean ± SEM; n = 3 to 5 biological replicates). The dotted red line indicates the level in untreated cells. *p < 0.05; ***p < 0.001 compared to control cells.

To investigate whether the induction of ΔNp63 protein in HaCaT cells stimulated with TGFβ for 24 h and the relative lack of alterations in FaDu and SCC-25 cells are related to transcriptional activation of *ΔNP63*, we performed RT-qPCR of cells treated with the same range of concentrations of TGFβ1 or TGFβ2 used above. Surprisingly, HaCaT cells showed lower levels of *ΔNP63* mRNA after 24 h treatment with concentrations of TGFβ1 and TGFβ2 that induced higher levels of ΔNp63 protein at this time (Fig. 2A). Comparable to the lack of changes in ΔNp63 protein, FaDu, and SCC-25 cells showed non-significant and variable changes in *ΔNP63* mRNA (Fig. 2B,C**)**. We repeated these experiments using 20 ng/ml TGFβ1 or TGFβ2 for 24 h in additional replicate experiments. Comparable effects were seen after exposure to either TGFβ1 or TGFβ2, and the combined data confirmed that 24 h treatment with TGFβ reduced *ΔNP63* mRNA in HaCaT cells (p = 7.18.10^–11^), whereas FaDu and SCC-25 cells showed no statistically significant changes in *ΔNP63* mRNA (Fig. 2D).

**Figure 3:**
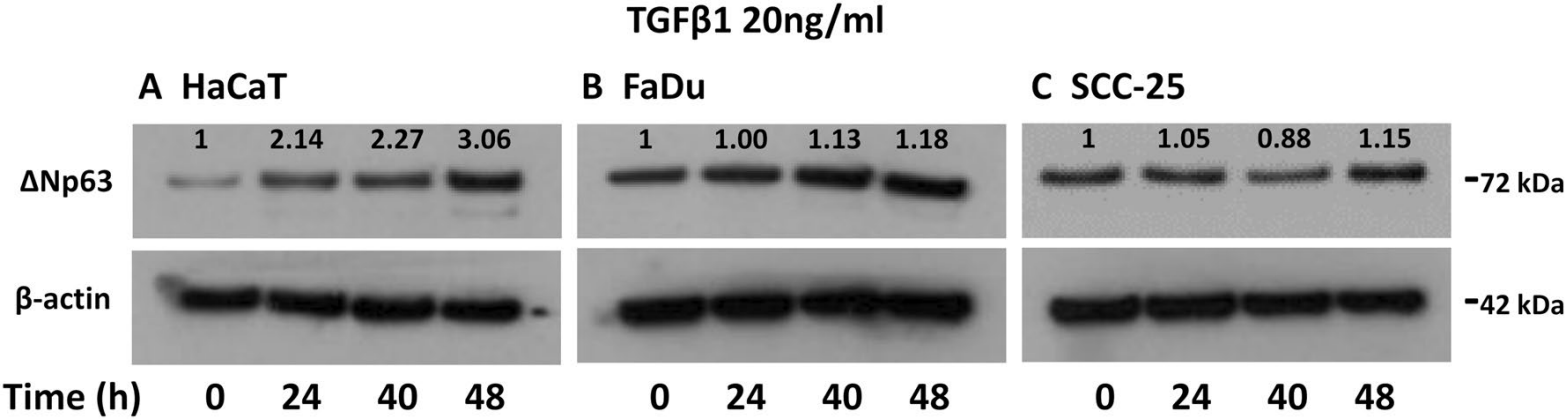
Cell-type dependent increase in ΔNp63 after prolonged TGFβ treatment. (**A**–**C**) Western blotting of ΔNp63 and β-actin as loading control in (**A**) HaCaT, (**B**) FaDu, and (**C**) SCC-25 cells treated with 20 ng/ml TGFβ1 for 0, 24, 40 or 48 h. Numbers above the ΔNp63 bands represent fold-change in band intensity compared to control cells, normalized to β-actin.

We subsequently extended the time course of TGFβ exposure to investigate whether the level of ΔNp63 protein was decreased at later times to parallel the decrease in mRNA levels at 24 h. However, ΔNp63 remained at an elevated level in HaCaT cells after 40 and 48 h of exposure, whereas the levels of ΔNp63 remained at control levels in FaDu and SCC-25 cells throughout (Fig. 3A–C).

### TGFβ induces biphasic induction/repression of *ΔNP63* and *TAP63* mRNAs in HaCaT cells

**Figure 4:**
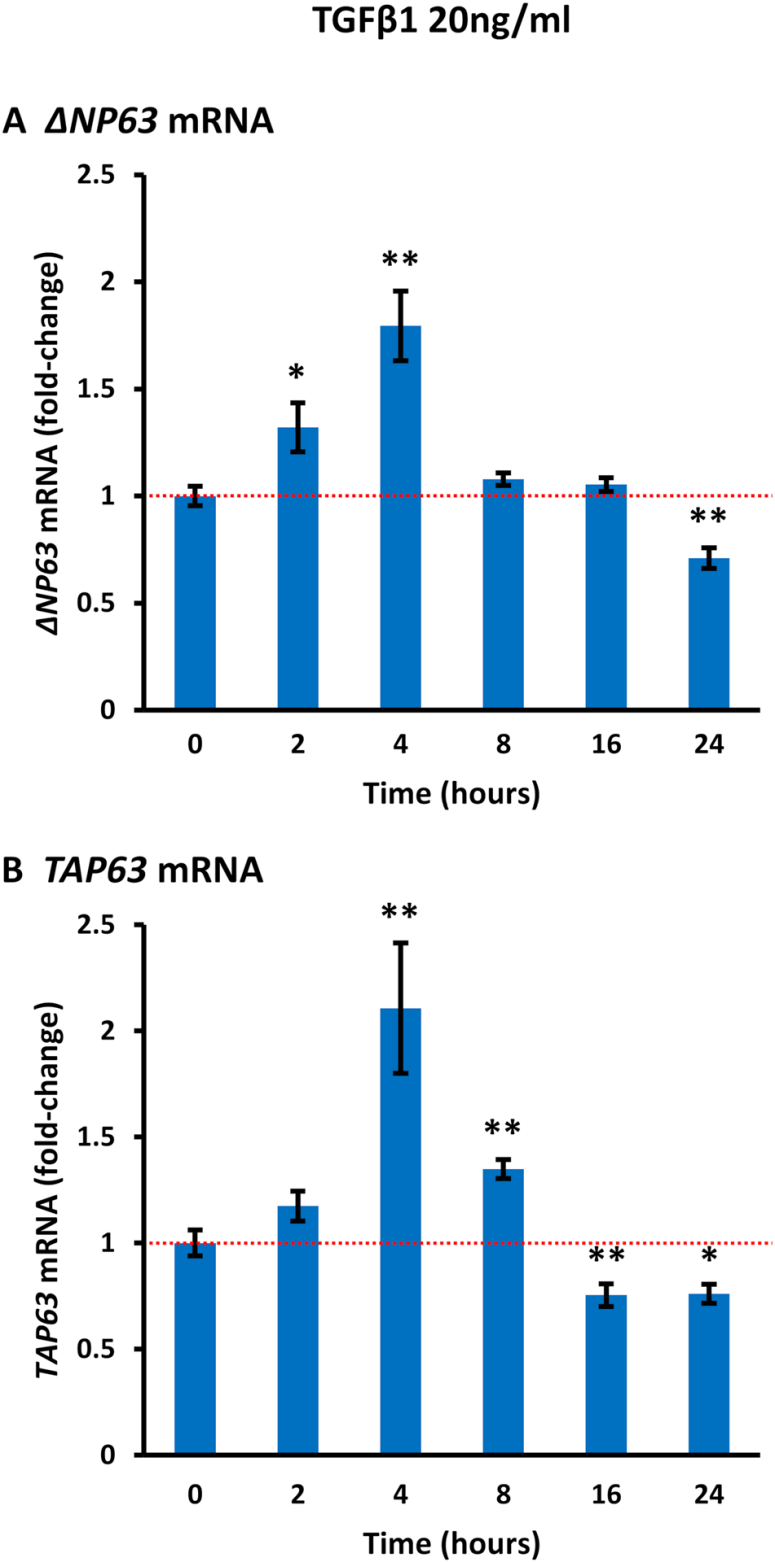
TGFβ causes biphasic induction and repression of *ΔNP63* and *TAP63* mRNAs in HaCaT cells. HaCaT cells were treated with 20 ng/ml TGFβ1 for the indicated times. RT-qPCR was performed for (**A**) *ΔNP63* or (**B**) *TAP63* mRNAs, using *ACTB* for normalization. The bar charts show mean fold-changes in mRNA levels at each time point (± SEM). The dotted red lines indicate the level in untreated cells. *p < 0.05; **p < 0.01.

These data indicated an inverse correlation between ΔNp63 protein and *ΔNP63* mRNA in HaCaT cells after treatment with TGFβ1 or TGFβ2. To further investigate the relationship between mRNA and protein levels, we performed a time course of *ΔNP63* mRNA in HaCaT cells. In view of the lack of protein or mRNA alterations in FaDu or SCC-25 cells, these were not included in this experiment. We also examined the levels of *TAP63* mRNA in HaCaT cells, which is detectable at the mRNA level at approximately 200-fold lower levels than *ΔNP63* mRNA but can be induced in these cells^42^. The results showed a biphasic response, with an initial induction of *ΔNP63* mRNA from 2 to 4 h, followed by decreased *ΔNP63* mRNA after 24 h exposure to 20 ng/ml TGFβ1 (Fig. 4A). *TAP63* mRNA showed similar kinetics of early induction followed by a decrease to lower levels than untreated cells at 24 h (Fig. 4B). Compatible with the very low levels of *TAP63* mRNA, TAp63 protein was not detected by Western blotting using a PAN-p63 antibody or an isoform-specific TAp63 antibody.

### TGFβ acts through TGFBR and SMAD signaling

**Figure 5:**
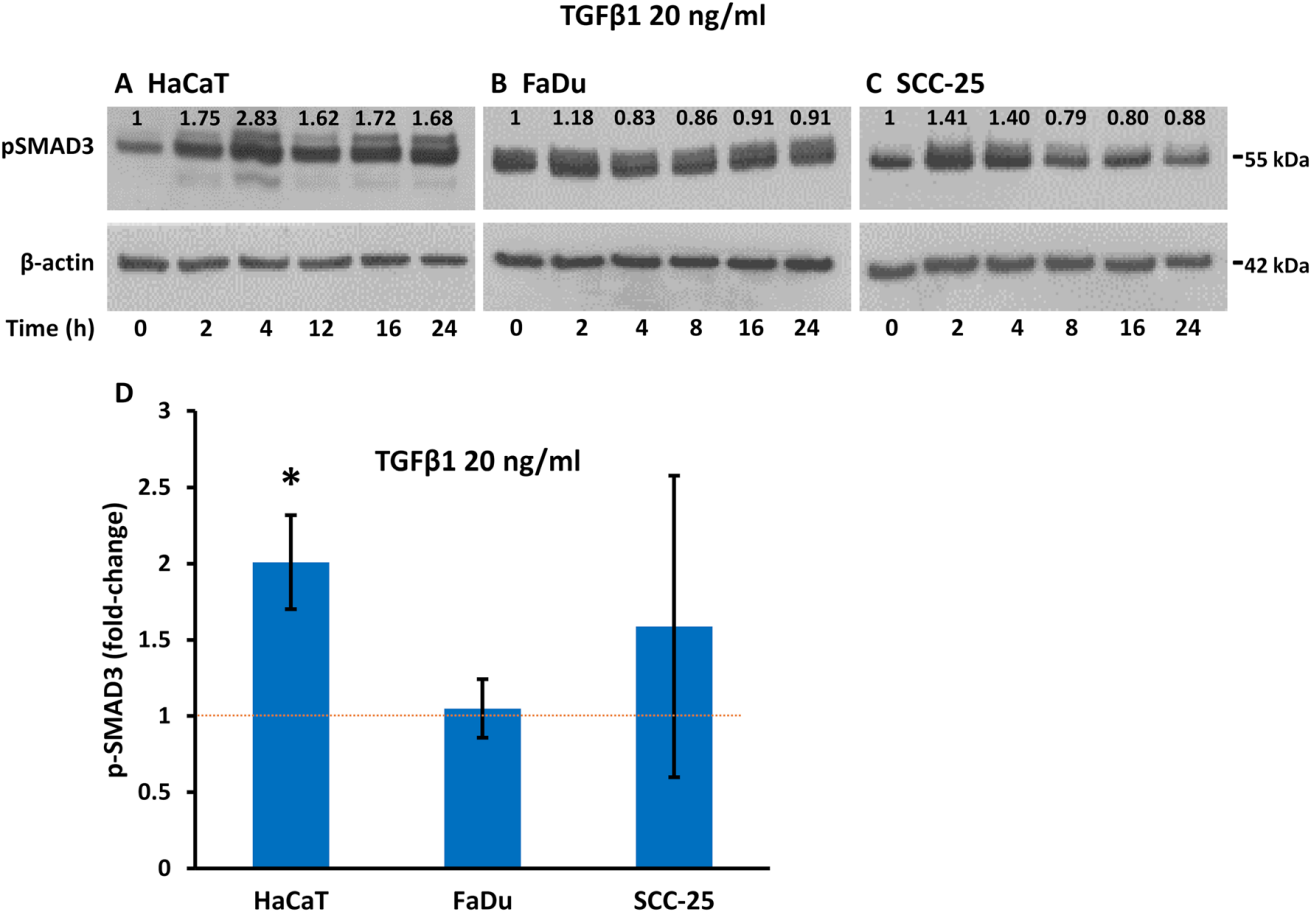
TGFβ induces cell-type dependent SMAD3 activation. (**A**–**C**) Western blotting of phosphorylated SMAD3 (p-SMAD3) and β-actin as loading control in (**A**) HaCaT, (**B**) FaDu, and (**C**) SCC-25 cells at the indicated times of treatment with 20 ng/ml TGFβ1. Numbers above the bands refer to fold-change in protein levels compared to control cells not exposed to TGFβ1, normalized to β-actin. (**D**) Average fold-change in p-SMAD3 in each cell line after treatment with 20 ng/ml TGFβ1 or TGFβ2 for 24 h compared to untreated cells (mean ± SEM; n = 3 to 4 biological replicates). The dotted red line indicates the level in untreated cells. *p < 0.05 compared to the corresponding control cells.

We next investigated whether the effects of TGFβ on p63 involve the canonical pathway through activation of SMAD2/3, and whether only HaCaT cells show SMAD activation to account for the cell-type differences in p63 regulation after TGFβ. For these experiments, we used measurements of SMAD3 phosphorylation, which correlates directly with canonical TGFβ signaling strength, whereas total SMAD levels do not correlate with TGFβ activity^3,7,45^. Treatment with TGFβ1 induced SMAD3 phosphorylation within 2 to 4 h in HaCaT and SCC-25 cells. The increased phosphorylation of SMAD3 was prolonged in HaCaT cells and was still evident after 24 h of continual treatment (Fig. 5A). Increased SMAD3 phosphorylation was not seen in FaDu cells (Fig. 5B) and was less altered and more transient in SCC-25 cells, where increased phosphorylation was lost at 8 h and longer times (Fig. 5C). Quantitation of replicate data from TGFβ1 and TGFβ2 stimulation revealed a twofold increase of p-SMAD3 in HaCaT cells 24 h after treatment, but no statistically significant change in p-SMAD3 in FaDu or SCC-25 cells (Fig. 5D). A longer time course showed prolonged p-SMAD3 activation in HaCaT cells stimulated with TGFβ1 for up to 48 h, but not in FaDu or SCC-25 cells under these conditions (see Supplementary Fig. S1 online).

**Figure 6:**
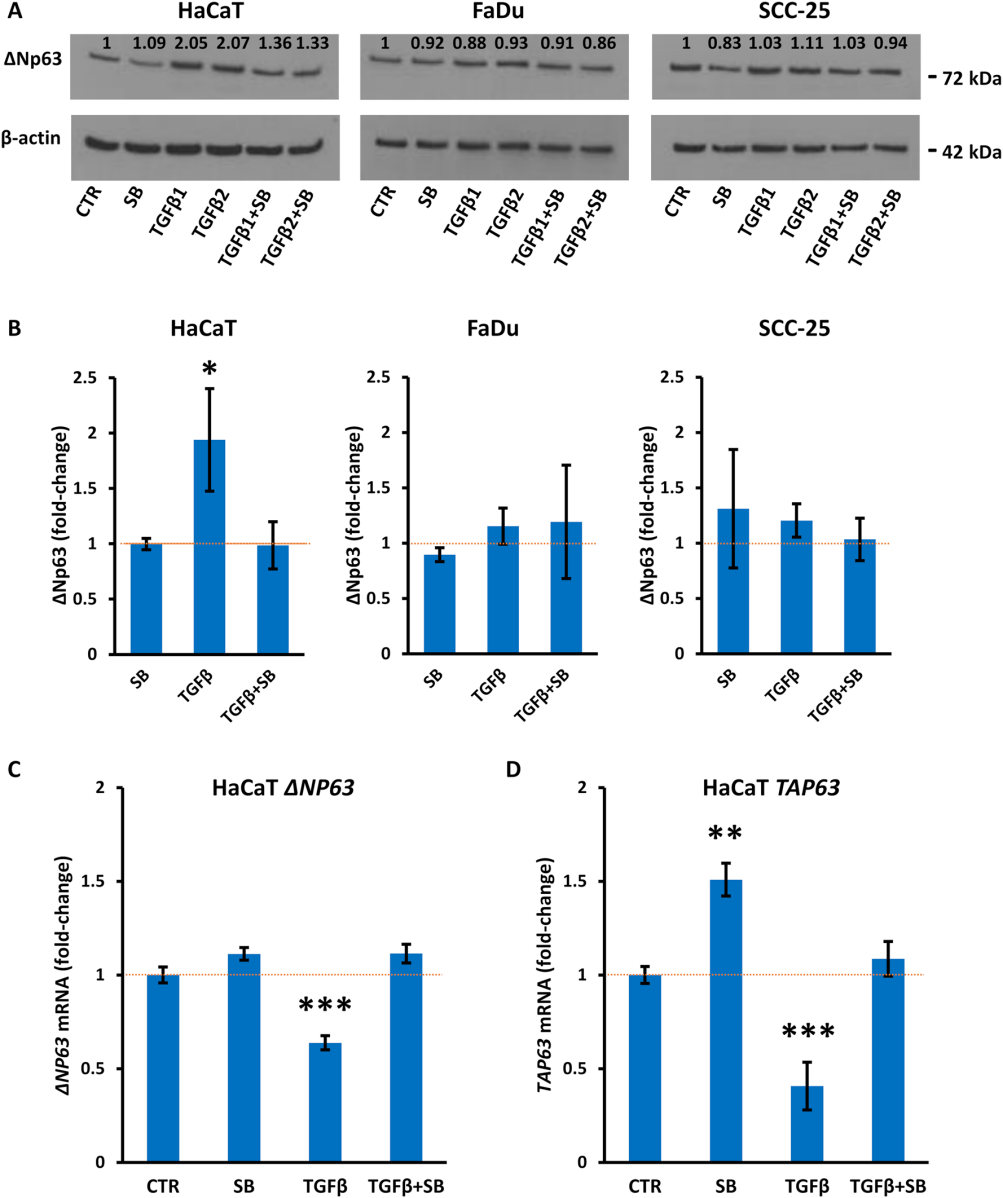
Differential regulation of p63 isoforms after inhibition of TGFBR. (**A**) Representative Western blots of ΔNp63 and β-actin as loading control in HaCaT, FaDu or SCC-25 control cells (CTR), cells exposed to SB431542 (SB) in the absence of TGFβ, exposed to TGFβ1 or TGFβ2 in the absence of inhibitor, or exposed to TGFβ1 or TGFβ2 in the presence of SB431542, each for 24 h. (**B**) Mean fold-changes in ΔNp63 protein normalized to β-actin protein levels (mean ± SEM; pooled data for TGFβ1 and TGFβ2 in the presence or absence of SB431542). (**C**) *ΔNP63* and (**D**) *TAP63* mRNA levels normalized to *ACTB* (mean ± SEM; n = 3 biological replicates). The dotted red line indicates the level in untreated cells. *p < 0.05; **p < 0.01; ***p < 0.001.

TGFβ1 and TGFβ2 act through TGFBR, a heterodimer of TGFBR1 and TGFBR2. To investigate whether endogenous TGFβ signaling is involved in the high basal levels of ΔNp63 in squamous cells, and to confirm the specificity of changes in p63 after TGFβ, cells were exposed to the TGFBR1 kinase inhibitor SB431542 in the presence or absence of TGFβ1 or TGFβ2. SB431542 decreased the effect of TGFβ on ΔNp63 levels in HaCaT cells after 24 h but did not influence the endogenous levels of ΔNp63 in the absence of added TGFβ (Fig. 6A,B). SB431542 did not change the levels of ΔNp63 in FaDu or SCC-25 cells in the presence or absence of TGFβ (Fig. 6A,B). We also found that SB431542 reduced the effect of TGFβ on SMAD3 phosphorylation in HaCaT cells but had minimal effects in FaDu or SCC-25 cells (see Supplementary Fig. S2 online).

At the transcriptional level, SB431542 did not influence *ΔNP63* mRNA levels in the absence of TGFβ but blocked the reduction in *ΔNP63* and *TAP63* mRNA levels after treatment with either TGFβ1 or TGFβ2 for 24 h (Fig. 6 C,D). SB431542 also increased the levels of *TAP63* mRNA in the absence of TGFβ (Fig. 6D).

### TGFβ induces morphological changes characteristic of EMT in HaCaT cells

As a final test of the cell-specific effects of TGFβ in squamous cells, we recorded the morphological changes that occur during TGFβ treatment. Cells were plated and allowed to attach overnight before treatment with 20 ng/ml TGFβ1. Replicate cells were pretreated with TGFBR inhibitor SB431542 for 1 h before exposure to TGFβ1 and maintained in the presence of SB431542 throughout the experiment. Cells were photographed at regular time points from 0 to 48 h. As expected from previous observations^46^, treatment of HaCaT cells with TGFβ caused EMT-like changes of cell enlargement and loss of cuboidal shape, together with cell spreading and loss of tight cell–cell connections at 24 h and longer. SB431542 inhibited these morphological changes. In comparison, identical treatment of FaDu and SCC-25 cells showed minimal changes in response to TGFβ (see Supplementary Fig. S3 online).

## Discussion

ΔNp63 is overexpressed in the majority of SCCs arising at different anatomical sites^15,16,18^, and is required for cell proliferation and survival in these cells^33,47^. Of clinical relevance, ΔNp63 levels correlate with poor differentiation, invasion, and aggressive tumors, and a correspondingly poor prognosis^18^. TGFβ aberrations are also common in SCC, altering signaling through mutations in TGFBR or TGFBR-regulated transcription factors (R-SMADs)^6^. There is substantial evidence for cross-talk between TGF-β and p63, with controversy over whether ΔNp63 is a positive or negative regulator of TGFβ signaling pathways, and whether TGFβ is a positive or negative regulator of ΔNp63, and information is lacking on the relative effects of TGFβ on TAp63 and ΔNp63 variants that have opposing oncogenic properties^9–12^.

Here, we systematically investigated the effects of TGFβ1 and TGFβ2 on p63 protein and mRNA isoforms in malignant and non-tumorigenic squamous cells. TGFβ1 and TGFβ2 possess context- and concentration-dependent functions^1–4^, and in our studies caused dose- and time-dependent upregulation of ΔNp63 protein in non-malignant HaCaT cells at 24 h. Surprisingly, there was a statistically significant decrease in *ΔNP63* mRNA at this time. However, the discrepancy is accounted for at least partly by the time course of *ΔNP63* mRNA changes, showing an initial increase at 2 to 4 h of TGFβ treatment before a decrease at 24 h. These data are similar to the early increase in *ΔNP63* mRNA levels in non-tumorigenic BEAS-2B lung epithelial cells and A431 epidermoid cells seen 2 to 4 h after stimulation with TGFβ but not later, accompanied by increased ΔNp63 protein at later times^36,37^. That FaDu cells showed no p63 response to TGFβ agrees with the lack of SMAD2/SMAD4 in these cells^36,48^, and that SCC-25 showed only a partial and transient response is in agreement with the minimal TGFβ-mediated effects in these cells, possibly related to their high endogenous levels of ETS1 that reduces TGFβ responsiveness^49–51^. Because all three cell lines are p53 mutant, differences in p53 signaling are unlikely to explain our results. Similarly, although TGFBR mutations are not uncommon in SCC, examination of the COSMIC cell line mutation data (https://cancer.sanger.ac.uk/cell_lines) shows that FaDu and SCC-25 do not contain *TGFBR1* or *TGFBR2* mutations, indicating that altered receptor activity due to mutation does not account for their differences in response.

Mechanistically, inhibition of TGFBR signaling blocked the effects of TGFβ, and changes to p63 protein and mRNA were associated with SMAD3 phosphorylation, indicating canonical R-SMAD pathway activation, consistent with some previous observations^36,38^. That there was no effect on p63 protein or mRNA in FaDu cells that lack canonical signaling implies that this is the major pathway involved in TGFβ-mediated effects in these cells. Moreover, the findings of increased p63 protein after TGFβ contradict previous observations of p63 degradation by TGFβ through non-canonical signaling and ubiquitin ligase-mediated p63 degradation^52,53^. Thus, the previous findings of disparate TGFβ actions on p63 likely reflect cell-type specific effects, such as activation of canonical *versus* non-canonical signaling, the cell-type specific expression of co-factors including IKKα that amplify the positive effects of TGFβ on p63^36^, and/or the presence and activation of specific E3-ligases for p63 degradation.

In addition to cell-type differences, the biphasic nature of *ΔNP63* and *TAP63* mRNA induction/repression that we uncovered may also help to explain previous discrepancies on the positive or negative regulation of p63 by TGFβ. This biphasic response suggests the involvement of at least two independent pathways. That *ΔNP63* and *TAP63* mRNAs increased within 2 to 4 h (Fig. 4), coinciding with SMAD3 activation (Fig. 5A), is compatible with transcriptional activation through two evolutionary conserved SMAD2/3 binding sites in the *ΔNP63* promoter^54^. Although relatively little is known about *TAP63* transcriptional regulation^9^, experiments in *Tgfb1*-null mice also found that *ΔNP63* and *TAP63* mRNA were co-regulated^40^, in agreement with our data of similar effects on both isoforms. In addition, we found that the baseline level of *TAP63* mRNA but not *ΔNP63* mRNA is influenced by SB431542, indicating differences in regulation of the two variants. Although the effect of SB431542 on *TAP63* mRNA in control cells may be due to inhibiting the effect of endogenous TGFβ in serum added to the tissue culture medium, these levels are low (1 -2 ng/ml) and TGF exists mainly as an inactive, latent form in cell culture^55^. Another possibility is that the effect of SB431542 on *TAP63* mRNA is related to inhibition of nodal or activin receptors, which are also inhibited by SB431542^56^, rather than TGFBR inhibition. Alternatively, given the high levels of endogenous ΔNp63 induced through other signaling pathways in squamous cells^9,10,47^, it may be that SB431542 is unable to overcome these intrinsic stimulatory factors. Given these findings, the mechanism of TGFβ regulation of *TAP63* will require further investigation.

The second phase of TGFβ regulation of p63 is the reduction of *TAP63* and *ΔNP63* mRNAs to below baseline levels within 24 h. Although we have not directly studied the mechanism(s) responsible, these data are compatible with TGFβ regulation of miRNAs that negatively regulate *TP63*, including miR-21, -22-3p, 30a-5p, 203a, and 222-3p^38,57^. These miRNAs bind to the shared 3’ UTR sequence of *TAP63* and *ΔNP63* mRNAs and would therefore reduce both transcripts, as seen in our data. Furthermore, many other miRNAs are implicated in *TP63* regulation and are themselves regulated by p63^9,18,58^. Which, if any, of these TGFβ- or p63-regulated miRNAs are involved in the biphasic regulation of *TP63* mRNAs in squamous cells will require further investigation. Our finding that ΔNp63 protein levels remain elevated up to 48 h after TGFβ stimulation despite reduced mRNA implies the existence of additional non-transcriptional regulation through yet unexplored mechanisms.

Our data have implications for the control of EMT in squamous cells, and imply that TGFβ in the presence of variable levels of p63 will produce intermediate or partial EMT phenotypes (pEMT), the direction of which will vary depending on endogenous p63 levels and on time- and cell type-dependent effects of TGFβ. pEMT and epithelial mesenchymal plasticity (EMP) are increasingly recognized as playing important roles in tumor cell migration, metastasis, and therapeutic resistance^59–61^. In particular, graded pEMT in SCC cells produces substantial intratumor cell heterogeneity, and the extent of the pEMT phenotype within individual tumors is influenced by TGFβ to determine the likelihood of metastasis and patient prognosis^62–64^.

In conclusion, we demonstrate that TGFβ produces complex time-dependent and cell-type dependent responses in squamous epithelial cells, with the most marked response in non-tumorigenic keratinocytes and lesser and variable responses in SCC cells, correlating with TGFβ pathway aberrations. The data help to explain the disparate and often opposite responses previously reported in different cell types examined at different times after stimulation. The heterogenous response that we highlight in SCC cells is likely to be caused by inherent genetic alterations that include differential utilization of epithelial-specific lineage transcription factors such as p63 itself, versus activation of EMT-inducing transcription factors including ZEB1/2, which define the SCC molecular subtypes^15,41^. In turn, the variable and time-dependent effects of TGFβ on p63 isoforms are likely to influence EMT and EMP in SCC, with the attendant clinical implications for tumor progression and response to therapy.

## Materials and methods

### Cell culture

HaCaT cells were obtained from the German Cancer Research Center (DKFZ; Heidelberg, Germany). These cells are spontaneously immortalized human keratinocytes that are non-tumorigenic and retain full squamous cell differentiation capacity. Malignant SCC cell lines, FaDu (human pharynx squamous cell carcinoma) and SCC-25 (human squamous cell carcinoma of the tongue), were obtained from the American Type Culture Collection (ATCC, Manassas, VI, USA). These are undifferentiated malignant SCC cell lines that do not undergo differentiation in response to confluency, and represent the mesenchymal (SCC-25) and the atypical (FaDu) molecular subtypes of SCC^41^. It should be noted that all cell lines have mutant p53 status (R282W and H179Y in HaCaT; R248L in FaDu; R209fs in SCC-25; https://p53.fr/tp53-database/the-tp53-cell-line-compendium). FaDu and HaCaT cells were maintained in high glucose Dulbecco’s modified Eagle’s medium (DMEM) with 10% fetal bovine serum (FBS), 1% sodium pyruvate, and penicillin/streptomycin (Gibco, Thermo Fisher Scientific, MA, USA) at 37 °C and 5% CO_2_. SCC-25 cells were cultured in DMEM/Nutrient Mixture F-12 (50:50) with 10% FBS, 0.4 μg/ml hydrocortisone (Lonza, Basel, Switzerland), 1% sodium pyruvate, and penicillin/streptomycin at 37 °C and 5% CO_2_. Under these culture conditions, the level of TGFβ is expected to be approximately 1 – 2 ng/ml and exists mainly as inactive latent TGFβ^55^. To avoid changes in p63 levels caused by density-dependent changes, depending on the experimental time and conditions, cells were plated at initial densities to reach approximately 80% confluency at the time of collection, and we were careful to ensure that full confluency was never reached.

### TGFβ treatment and inhibition

All chemicals and growth factors were obtained from Sigma-Aldrich (St Louis, MO, USA) unless stated otherwise. TGFβ1 (SKU 100–21) and TGFβ2 (SKU 100-35B) were purchased from Peprotech (supplied by Baria, Prague, Czech Republic). Compounds were dissolved according to the manufacturer’s instructions and controls were performed using the highest volume of solute. Cells were initially analyzed by Western blotting after 24 h incubation with a range of concentrations of either TGFβ1 or TGFβ2. From these experiments, 20 ng/ml TGFβ was selected for further experiments. We used the low molecular weight inhibitor SB431542 (S4317, Sigma-Aldrich) to inhibit TGFBR signaling. SB431542 is a competitive ATP binding site kinase inhibitor that inhibits TGFBR1 and the activin and nodal receptors, but not BMP or non-canonical signaling pathways^56,65^. Cells were pre-incubated for 1 h with 10 µM SB431542 before adding TGFβ1/2^8^.

### SDS-PAGE and Western blot analysis

The detailed procedure is provided elsewhere^42^. In brief, cells were lysed in 150 mM NaCl, 1% NP-40, 50 mM Tris pH 8.0 containing protease and phosphatase inhibitors (Thermo Fisher Scientific, Waltham, MA, USA), and 25—40 µg total proteins were separated on 10% polyacrylamide gels and transferred onto nitrocellulose. The membranes were cut into upper and lower portions; the upper membrane strips were incubated overnight at 4 °C with mouse monoclonal antibodies to ΔNp63 (clone ΔNp63-1.1, Moravian Biotechnology, Brno, Czech Republic) or TAp63 (clone TAp63-4.1, Moravian Biotechnology)^42^, or with rabbit monoclonal antibody to p-Smad3 (clone EP823Y, Abcam, Cambridge, UK) that recognizes Smad3 phosphorylated at Ser423/425^8^. The lower membrane strip from the same gel was probed for β-actin (mouse monoclonal clone C4, Santa Cruz, Dallas, TX, USA) as a loading control for densitometry normalization. After washing and incubation with peroxidase-labeled secondary antibodies (Jackson Immunoresearch, West Grove, PA, USA), bands were visualized using enhanced chemiluminescence (ECL, Amersham Pharmacia Biotech, Bucks, UK). ImageJ (imageJ.net/ij/index/html) was used for densitometry measurements.

### RNA isolation and reverse transcription quantitative PCR (RT-qPCR)

Total RNA was isolated using TRIzol and quantified using A260 measurements (NanoDrop, Thermo FisherScientific). To ensure equal cell numbers were analyzed in each reaction, 500 ng RNA from each sample were reverse transcribed using High-Capacity cDNA Reverse Transcription (Applied Biosystems, Thermo Fisher Scientific). Primers for *ΔNP63, TAP63,* and *ACTB* (β-actin)^42^ (Generi Biotech, Hradec Kralove, Czech Republic) were used for quantitative PCR on a Fast Real-Time PCR System with SYBR Green (Applied Biosystems): 95 °C for 3 min, 50 cycles of 95 °C for 5 s and 60 °C for 25 s. Each cDNA sample was analyzed in technical triplicates of independent samples (biological replicates). Cycle threshold (Ct) values were transformed into relative mRNA levels^66^ and normalized to *ACTB* to account for any differences in results due to differences in the amount of RNA analyzed in different experimental procedures and for other technical factors.

### Cell morphology

Cell morphology after TGFβ treatment in the presence or absence of SB431542 was monitored using phase contrast microscopy to investigate TGFβ-induced changes that typify EMT (loss of cuboidal epithelial shape and reduced cell–cell contact).

### Statistical analysis

Data were tested for normality of distribution using Shapiro-Wilks tests (significance level (α) = 0.05), with skewness and kurtosis examination (https://www.statskingdom.com/shapiro-wilk-test-calculator.html). Results are presented as mean ± SEM of biological replicates. Statistical significance (p < 0.05) was determined using unpaired 2-tailed t-tests.

### Supplementary Information

Supplementary Figure 1.

Supplementary Figure 2.

## Data Availability

All data generated or analyzed during this study are included in this published article and its supplementary information files.

## Abbreviations

BMP: Bone morphogenic protein
CSC: Cancer stem cell
EMP: Epithelial-to-mesenchymal plasticity
EMT: Epithelial-to-mesenchymal transition
pEMT: Partial EMT
R-SMAD: Receptor activated SMAD
SCC: Squamous cell carcinoma
TGFβ: Transforming growth factor beta
TGFBR: TGFβ receptor

## References

[1] Budi EH, Schaub JR, Decaris M, Turner S, Derynck R (2021). TGF-β as a driver of fibrosis: Physiological roles and therapeutic opportunities. J. Pathol..

[2] David CJ, Massagué J (2018). Contextual determinants of TGFβ action in development, immunity and cancer. Nat. Rev. Mol. Cell Biol..

[3] Seoane J, Gomis RR (2017). TGF-β family signaling in tumor suppression and cancer progression. Cold Spring Harb. Perspect. Biol..

[4] Zhang Y, Alexander PB, Wang X-F (2017). TGF-β Family Signaling in the Control of Cell Proliferation and Survival. Cold Spring Harb. Perspect. Biol..

[5] Mani SA (2008). The epithelial-mesenchymal transition generates cells with properties of stem cells. Cell.

[6] Pang X, Tang Y-L, Liang X-H (2018). Transforming growth factor-β signaling in head and neck squamous cell carcinoma: Insights into cellular responses. Oncol. Lett..

[7] Cammareri P (2016). Inactivation of TGFβ receptors in stem cells drives cutaneous squamous cell carcinoma. Nat. Commun..

[8] Rose AM (2018). Reduced SMAD2/3 activation independently predicts increased depth of human cutaneous squamous cell carcinoma. Oncotarget.

[9] Pokorná Z, Vysloužil J, Hrabal V, Vojtěšek B, Coates PJ (2021). The foggy world(s) of p63 isoform regulation in normal cells and cancer. J. Pathol..

[10] Fisher ML, Balinth S, Mills AA (2023). ΔNp63α in cancer: importance and therapeutic opportunities. Trends Cell Biol..

[11] Crum CP, McKeon FD (2010). p63 in epithelial survival, germ cell surveillance, and neoplasia. Annu. Rev. Pathol..

[12] Orzol P (2015). The diverse oncogenic and tumour suppressor roles of p63 and p73 in cancer: A review by cancer site. Histol. Histopathol..

[13] Blanpain C, Fuchs E (2007). p63: revving up epithelial stem-cell potential. Nat. Cell Biol..

[14] Melino G, Memmi EM, Pelicci PG, Bernassola F (2015). Maintaining epithelial stemness with p63. Sci. Signal..

[15] Campbell JD (2018). Genomic, pathway network, and immunologic features distinguishing squamous carcinomas. Cell Rep..

[16] Dotto GP, Rustgi AK (2016). Squamous cell cancers: A unified perspective on biology and genetics. Cancer Cell.

[17] Nekulova M, Holcakova J, Coates P, Vojtesek B (2011). The role of p63 in cancer, stem cells and cancer stem cells. Cell. Mol. Biol. Lett..

[18] Moses MA (2019). Molecular mechanisms of p63-mediated squamous cancer pathogenesis. Int. J. Mol. Sci..

[19] Loljung L (2014). High expression of p63 is correlated to poor prognosis in squamous cell carcinoma of the tongue. *J. Oral Pathol. Med. Off. Publ. Int*. Assoc. Oral Pathol. Am. Acad. Oral Pathol..

[20] Bretz AC (2016). ΔNp63 activates the Fanconi anemia DNA repair pathway and limits the efficacy of cisplatin treatment in squamous cell carcinoma. Nucleic Acids Res..

[21] Lo Muzio L (2005). p63 overexpression associates with poor prognosis in head and neck squamous cell carcinoma. Hum. Pathol..

[22] Moergel M, Abt E, Stockinger M, Kunkel M (2010). Overexpression of p63 is associated with radiation resistance and prognosis in oral squamous cell carcinoma. Oral Oncol..

[23] Thurfjell N (2005). Endogenous p63 acts as a survival factor for tumour cells of SCCHN origin. Int. J. Mol. Med..

[24] Tran MN (2013). The p63 protein isoform ΔNp63α inhibits epithelial-mesenchymal transition in human bladder cancer cells: role of MIR-205. J. Biol. Chem..

[25] Olsen JR (2013). p63 attenuates epithelial to mesenchymal potential in an experimental prostate cell model. PloS ONE.

[26] Mehrazarin S (2015). The p63 gene is regulated by grainyhead-like 2 (GRHL2) through reciprocal feedback and determines the epithelial phenotype in human keratinocytes. J. Biol. Chem..

[27] Yoh KE (2016). Repression of p63 and induction of EMT by mutant Ras in mammary epithelial cells. Proc. Natl. Acad. Sci. USA.

[28] Citro S (2019). Synergistic antitumour activity of HDAC inhibitor SAHA and EGFR inhibitor gefitinib in head and neck cancer: a key role for ΔNp63α. Br. J. Cancer.

[29] Min S (2020). p63 and its target follistatin maintain salivary gland stem/progenitor cell function through TGF-β/activin signaling. Iscience.

[30] Testoni B (2006). Identification of new p63 targets in human keratinocytes. Cell Cycle Georget. Tex.

[31] Sundqvist A (2020). TGFβ and EGF signaling orchestrates the AP-1- and p63 transcriptional regulation of breast cancer invasiveness. Oncogene.

[32] Oh J-E, Kim RH, Shin K-H, Park N-H, Kang MK (2011). DeltaNp63α protein triggers epithelial-mesenchymal transition and confers stem cell properties in normal human keratinocytes. J. Biol. Chem..

[33] Abraham CG (2018). ΔNp63α suppresses TGFB2 expression and RHOA activity to drive cell proliferation in squamous cell carcinomas. Cell Rep..

[34] Adorno M (2009). A Mutant-p53/Smad complex opposes p63 to empower TGFbeta-induced metastasis. Cell.

[35] Vasilaki E (2016). Ras and TGF-β signaling enhance cancer progression by promoting the ΔNp63 transcriptional program. Sci. Signal..

[36] Fukunishi N (2010). Induction of ΔNp63 by the newly identified keratinocyte-specific transforming growth factor β Signaling Pathway with Smad2 and IκB Kinase α in squamous cell carcinoma. Neoplasia N. Y. N.

[37] Murata K (2007). p63 - Key molecule in the early phase of epithelial abnormality in idiopathic pulmonary fibrosis. Exp. Mol. Pathol..

[38] Bui NHB (2020). Spatiotemporal Regulation of ΔNp63 by TGFβ-regulated miRNAs Is essential for cancer metastasis. Cancer Res..

[39] Mou H (2016). Dual SMAD Signaling Inhibition Enables Long-Term Expansion of Diverse Epithelial Basal Cells. Cell Stem Cell.

[40] Chalmers FE, Dusold JE, Shaik JA, Walsh HA, Glick AB (2022). Targeted deletion of TGFβ1 in basal keratinocytes causes profound defects in stratified squamous epithelia and aberrant melanocyte migration. Dev. Biol..

[41] Walter V (2013). Molecular subtypes in head and neck cancer exhibit distinct patterns of chromosomal gain and loss of canonical cancer genes. PloS One.

[42] Pokorna Z, Hrabal V, Tichy V, Vojtesek B, Coates PJ (2022). DNA Demethylation Switches Oncogenic ΔNp63 to Tumor Suppressive TAp63 in Squamous Cell Carcinoma. Front. Oncol..

[43] Jennings JC, Mohan S, Linkhart TA, Widstrom R, Baylink DJ (1988). Comparison of the biological actions of TGF beta-1 and TGF beta-2: differential activity in endothelial cells. J. Cell. Physiol..

[44] O’Sullivan MJ (2021). In well-differentiated primary human bronchial epithelial cells, TGF-β1 and TGF-β2 induce expression of furin. Am. J. Physiol. Lung Cell. Mol. Physiol..

[45] Schmierer B, Hill CS (2007). TGFbeta-SMAD signal transduction: molecular specificity and functional flexibility. Nat. Rev. Mol. Cell Biol..

[46] Davies M (2005). Induction of an epithelial to mesenchymal transition in human immortal and malignant keratinocytes by TGF-beta1 involves MAPK, Smad and AP-1 signalling pathways. J. Cell. Biochem..

[47] Pokorna Z, Vyslouzil J, Vojtesek B, Coates PJ (2022). Identifying pathways regulating the oncogenic p53 family member ΔNp63 provides therapeutic avenues for squamous cell carcinoma. Cell. Mol. Biol. Lett..

[48] Hummer BT, Bartlett C, Henry E, Weissman BE (2003). Expression of Smad4 in the FaDu cell line partially restores TGF-beta growth inhibition but is not sufficient to regulate fibronectin expression or suppress tumorigenicity. J. Cell. Physiol..

[49] Dahler AL, Cavanagh LL, Saunders NA (2001). Suppression of keratinocyte growth and differentiation by transforming growth factor beta1 involves multiple signaling pathways. J. Invest. Dermatol..

[50] Ingruber J (2022). EMT-related transcription factors and protein stabilization mechanisms involvement in cadherin switch of head and neck squamous cell carcinoma. Exp. Cell Res..

[51] Gluck C (2019). Molecular dissection of the oncogenic role of ETS1 in the mesenchymal subtypes of head and neck squamous cell carcinoma. PLoS Genet..

[52] Cherukuri P (2012). Phosphorylation of ΔNp63α via a novel TGFβ/ALK5 signaling mechanism mediates the anti-clonogenic effects of TGFβ. PloS One.

[53] Niu M (2021). Noncanonical TGF-β signaling leads to FBXO3-mediated degradation of ΔNp63α promoting breast cancer metastasis and poor clinical prognosis. PLoS Biol..

[54] Laronda MM (2013). Diethylstilbestrol induces vaginal adenosis by disrupting SMAD/RUNX1-mediated cell fate decision in the Müllerian duct epithelium. Dev. Biol..

[55] Oida T, Weiner HL (2010). Depletion of TGF-β from fetal bovine serum. J. Immunol. Methods.

[56] Inman GJ (2002). SB-431542 is a potent and specific inhibitor of transforming growth factor-beta superfamily type I activin receptor-like kinase (ALK) receptors ALK4, ALK5, and ALK7. Mol. Pharmacol..

[57] Wang T (2012). TGF-β-induced miR-21 negatively regulates the antiproliferative activity but has no effect on EMT of TGF-β in HaCaT cells. Int. J. Biochem. Cell Biol..

[58] Candi E, Amelio I, Agostini M, Melino G (2015). MicroRNAs and p63 in epithelial stemness. Cell Death Differ..

[59] Brabletz S, Schuhwerk H, Brabletz T, Stemmler MP (2021). Dynamic EMT: a multi-tool for tumor progression. EMBO J..

[60] Haerinck J, Goossens S, Berx G (2023). The epithelial-mesenchymal plasticity landscape: principles of design and mechanisms of regulation. Nat. Rev. Genet..

[61] Lambert AW (2022). ΔNp63/p73 drive metastatic colonization by controlling a regenerative epithelial stem cell program in quasi-mesenchymal cancer stem cells. Dev. Cell.

[62] Tyler M, Tirosh I (2021). Decoupling epithelial-mesenchymal transitions from stromal profiles by integrative expression analysis. Nat. Commun..

[63] Jung AR, Jung C-H, Noh JK, Lee YC, Eun Y-G (2020). Epithelial-mesenchymal transition gene signature is associated with prognosis and tumor microenvironment in head and neck squamous cell carcinoma. Sci. Rep..

[64] Kisoda S (2022). The role of partial-EMT in the progression of head and neck squamous cell carcinoma. J. Oral Biosci..

[65] Callahan JF (2002). Identification of novel inhibitors of the transforming growth factor beta1 (TGF-beta1) type 1 receptor (ALK5). J. Med. Chem..

[66] Pfaffl MW (2001). A new mathematical model for relative quantification in real-time RT-PCR. Nucleic Acids Res..

