# Supplementary material for: Complex and variable regulation of ΔNp63 and TAp63 by TGFβ has implications for the dynamics of squamous cell epithelial to mesenchymal transition
Source: Sci Rep. 2024 Mar 27;14:7304. doi: 10.1038/s41598-024-57895-1 (PMC10973453; doi:10.1038/s41598-024-57895-1)
Supplement: Supplementary file 1 — Supplementary Figure 1. [file 41598_2024_57895_MOESM1_ESM.pdf]

Original images for Fig. S1
HaCaT, FaDu or SCC-25 cells treated with TGFβ1 .
Westerns for p-SMAD3 and β-actin

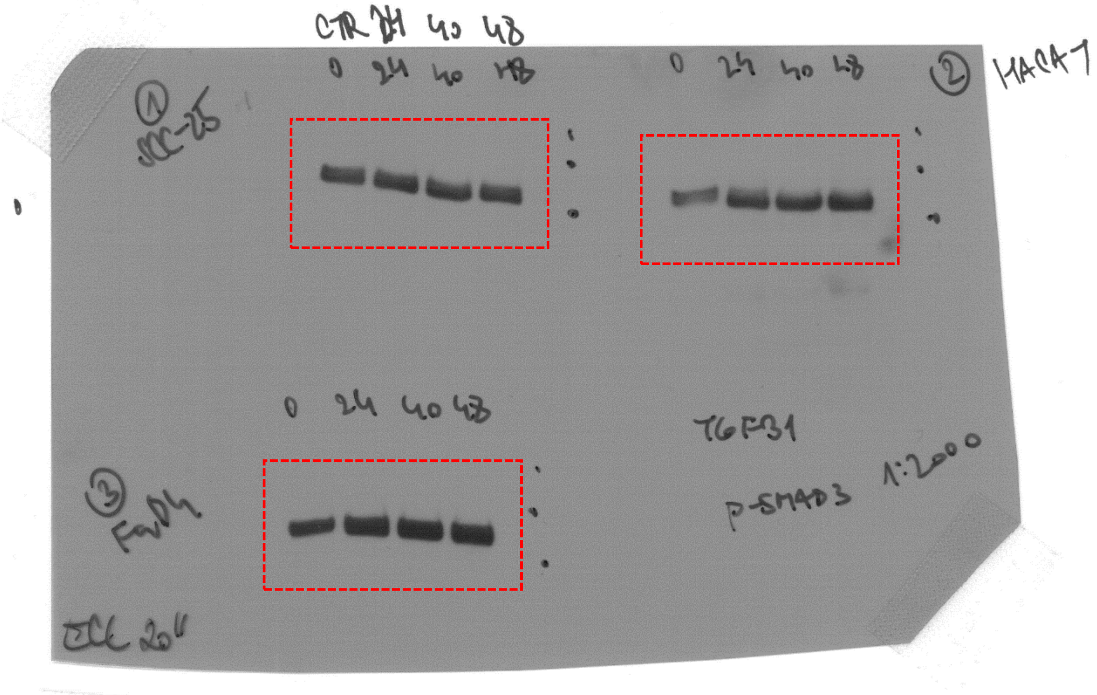

SCC-25

HaCaT

FaDu

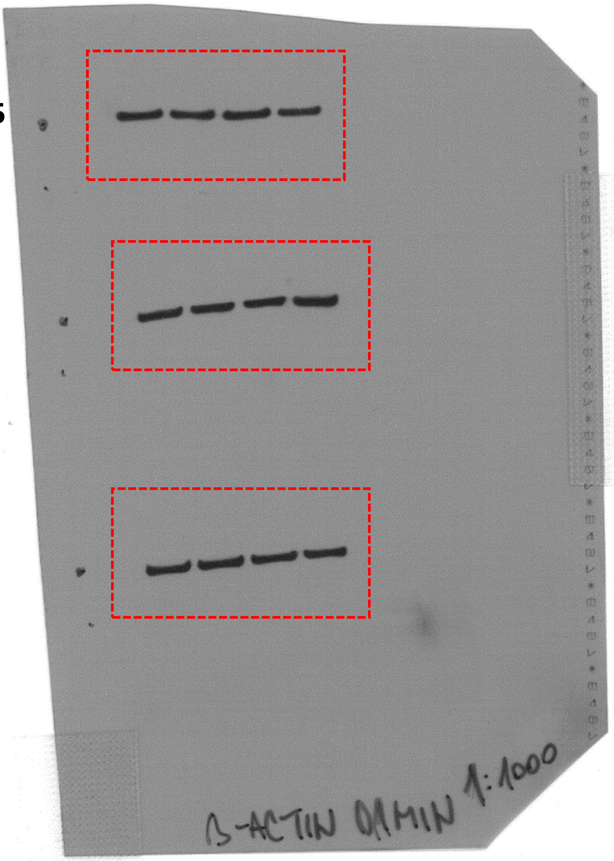

Original images for Fig. S2
HaCaT cells, control, treated with SB, treated with TGFβ1, or with TGFβ1 + SB.
Western blot for p-SMAD3

HaCaT

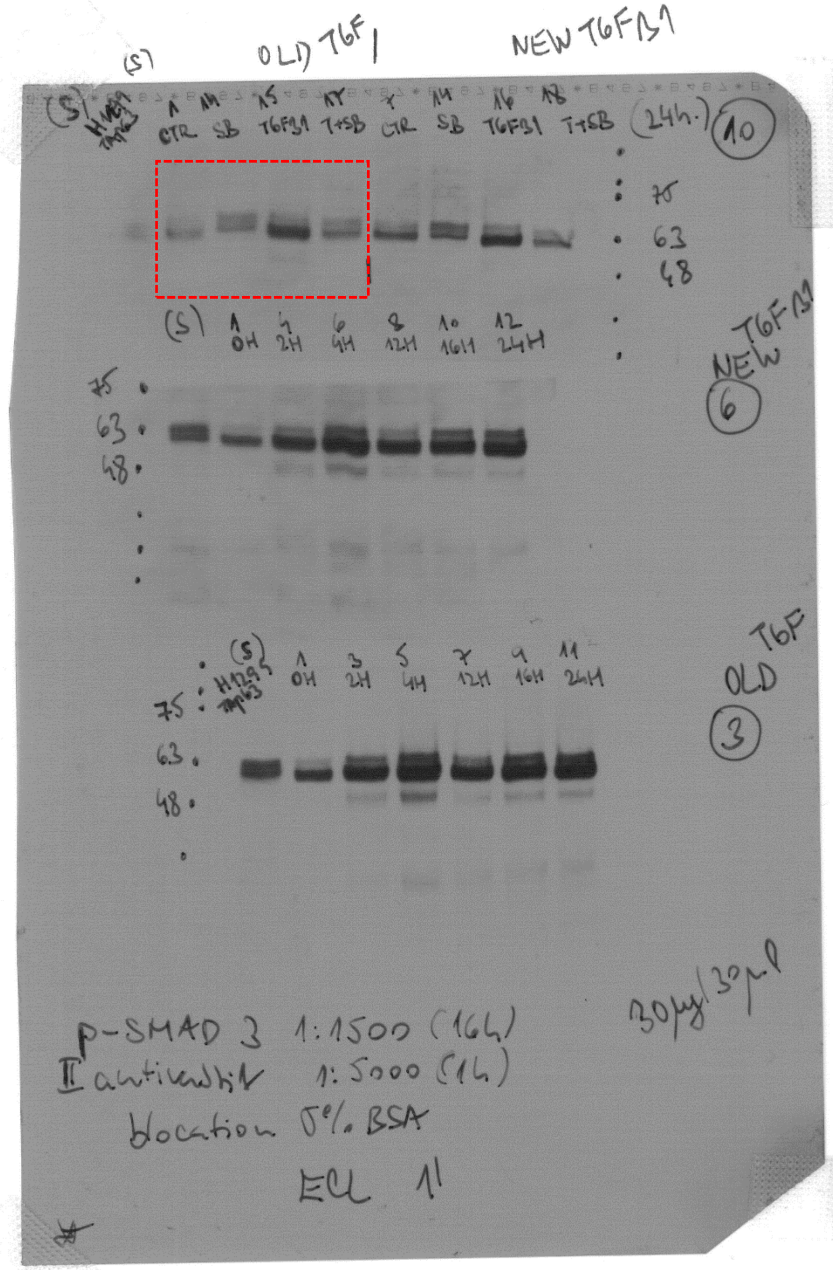

Original images for Fig. S2
FaDu or SCC-25 cells, control, treated with SB, treated with TGFβ1, or with TGFβ1 + SB.
Western blot for p-SMAD3

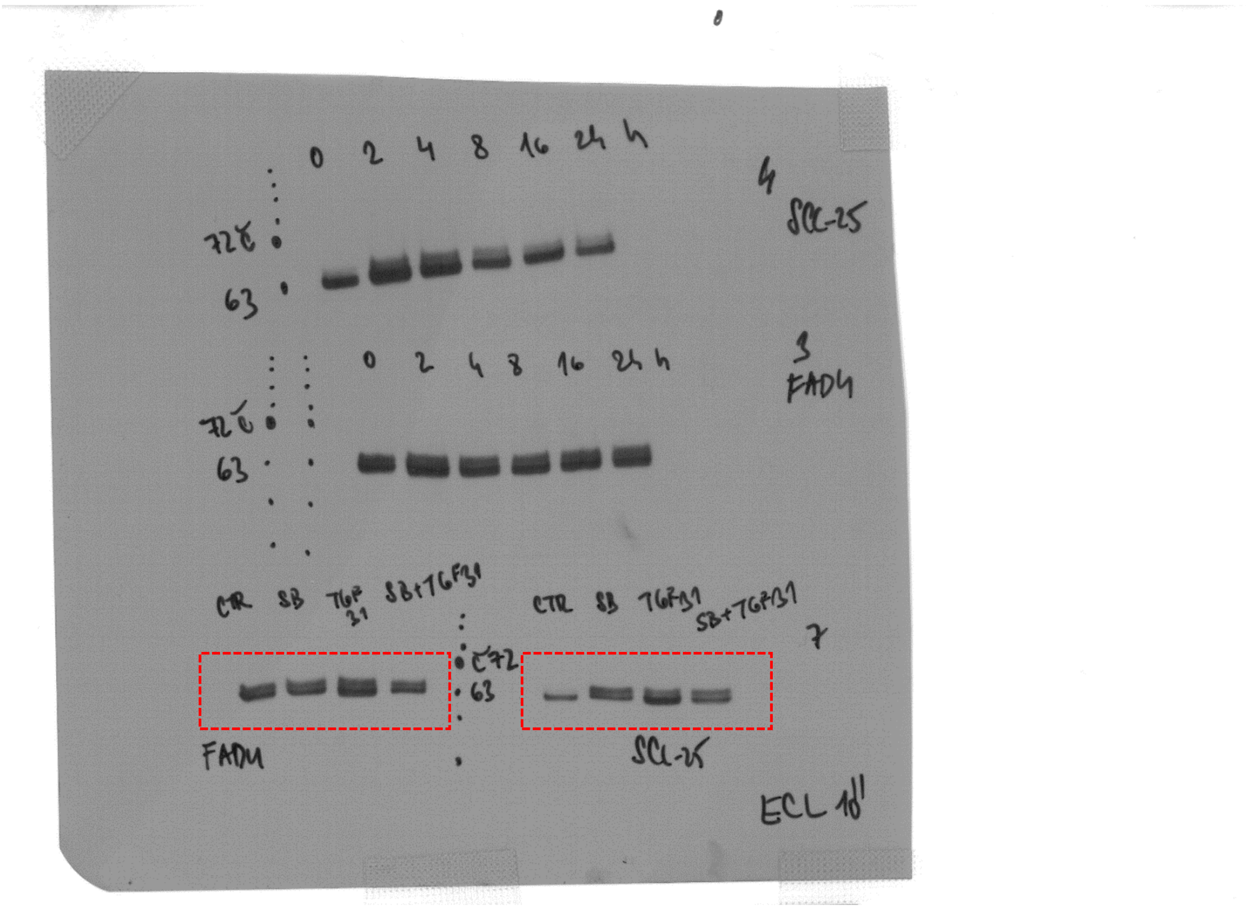

Original images for Fig. S2
HaCaT cells, control, treated with SB, treated with TGFβ1, or with TGFβ1 + SB.
Western blot for β-actin

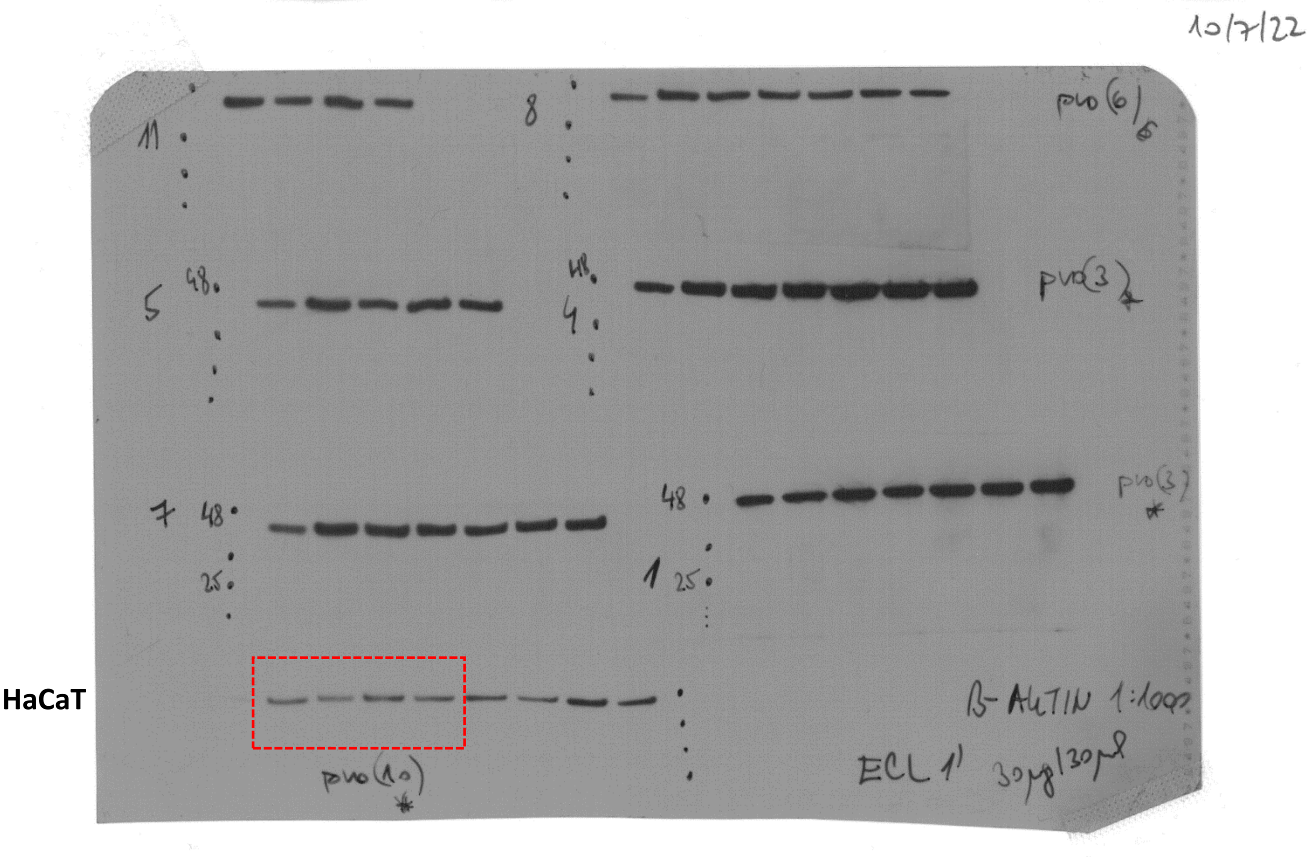

Original images for Fig. S2
FaDu or SCC-25 cells, control, treated with SB, treated with TGFβ1, or with TGFβ1 + SB.
Western blot for β-actin

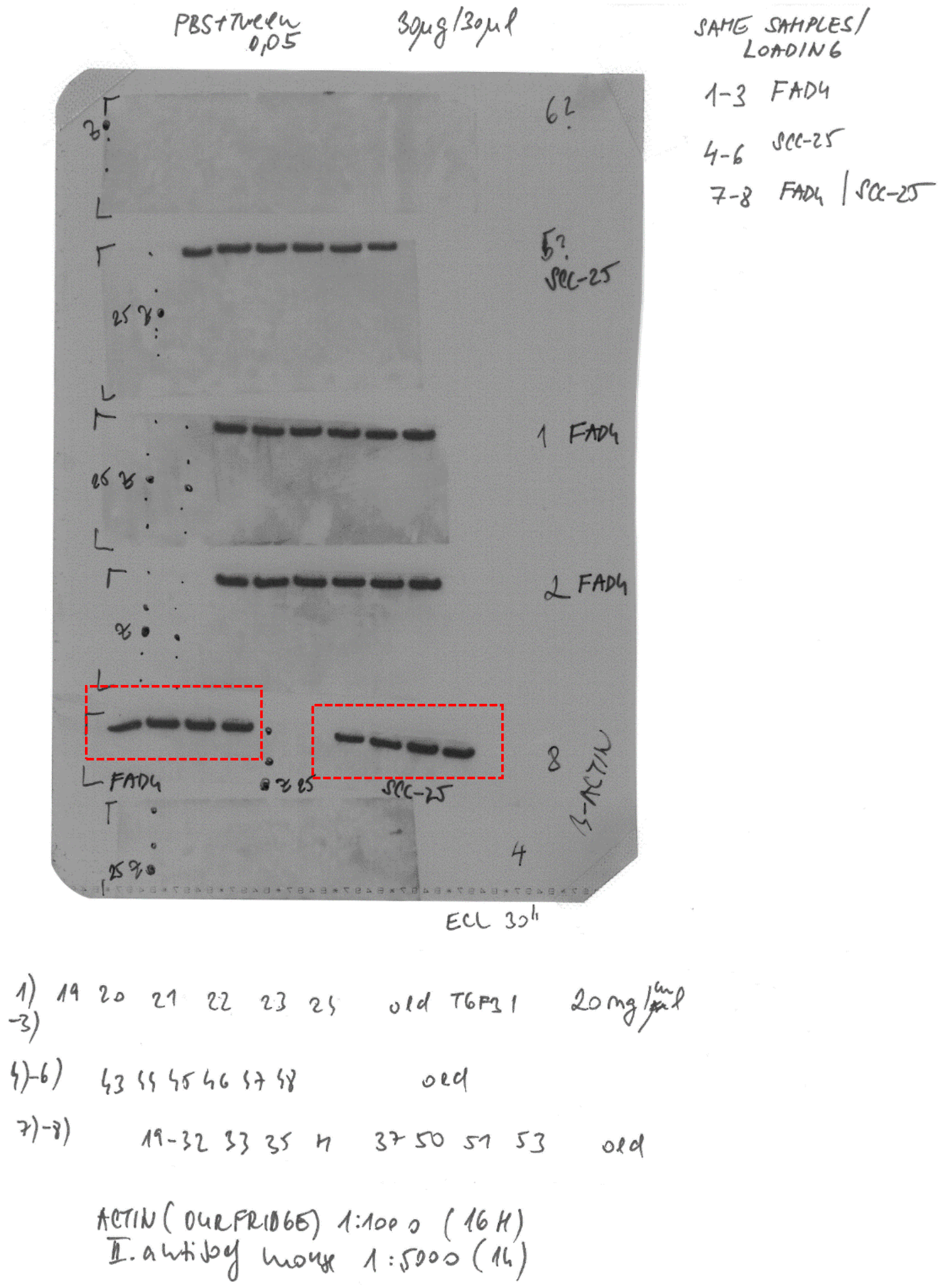

Original images for Fig. S3 Scale Bar = 400 µm

HaCaT cells

Control (0 h)

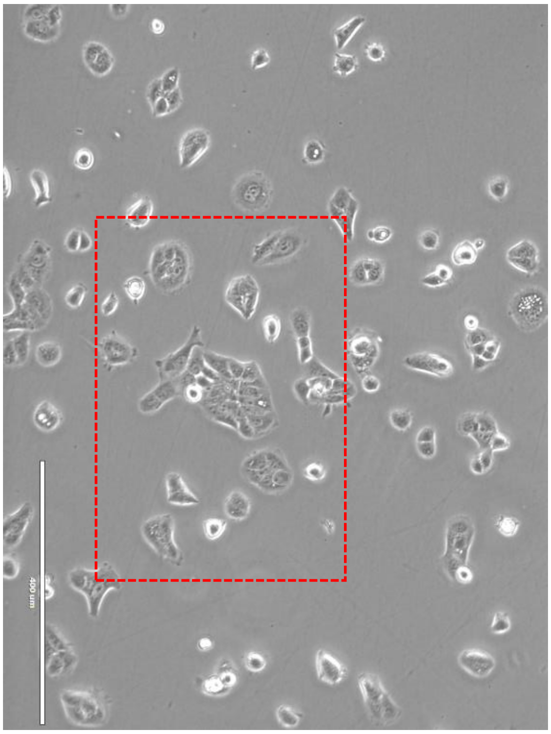

TGFβ (36 h)

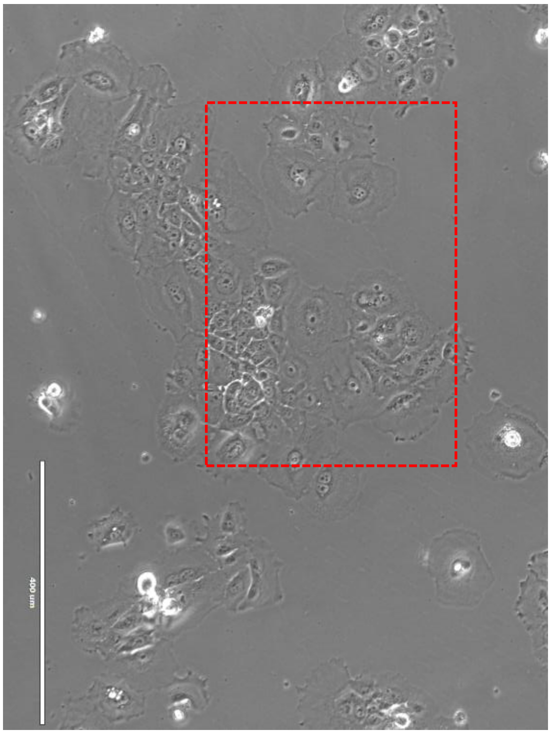

TGFβ + SB (36 h)

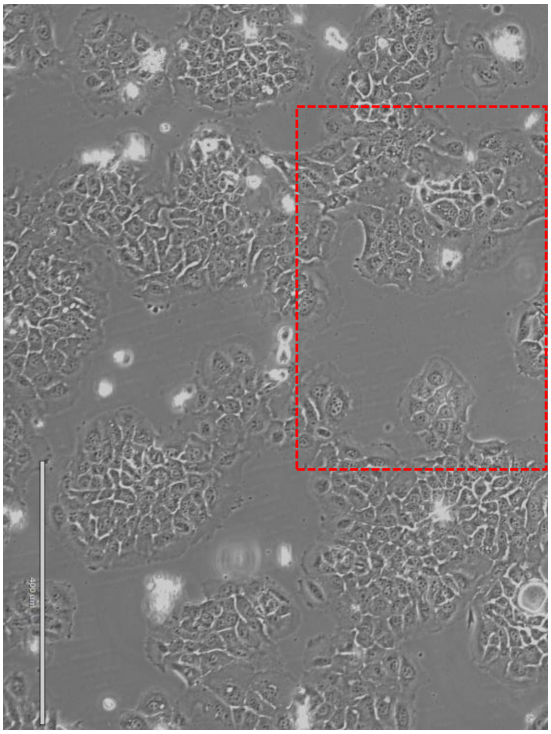

Original images for Fig. S3 Scale Bar = 400 µm

FaDu cells

Control (0 h)

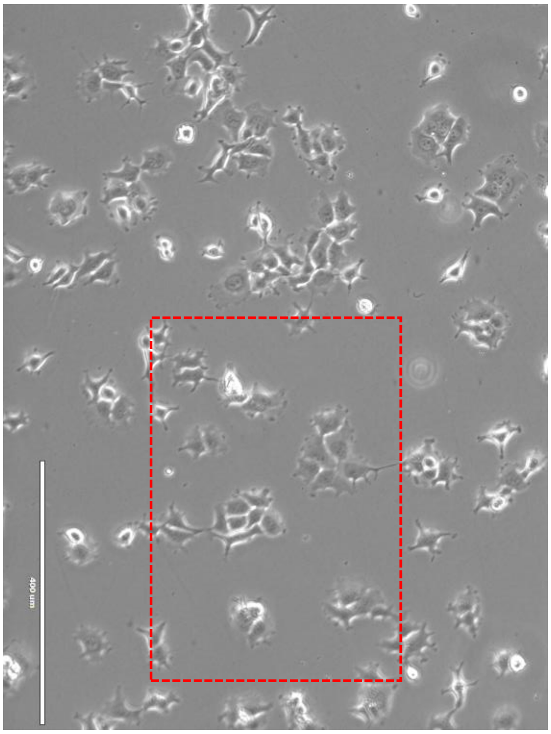

TGFβ (36 h)

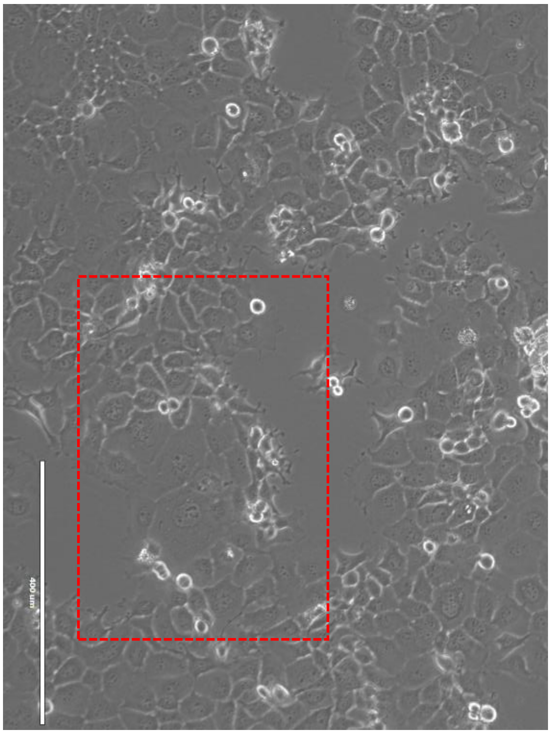

TGFβ + SB (36 h)

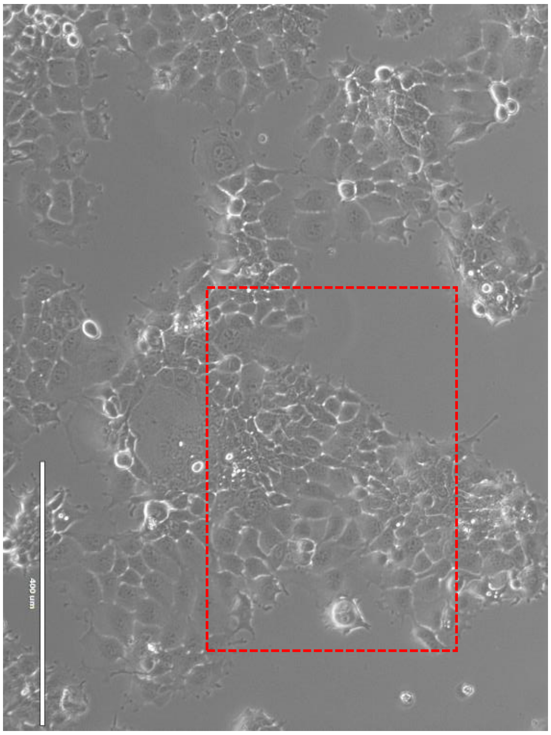

Original images for Fig. S3 Scale Bar = 400 µm

FaDu cells

Control (0 h)

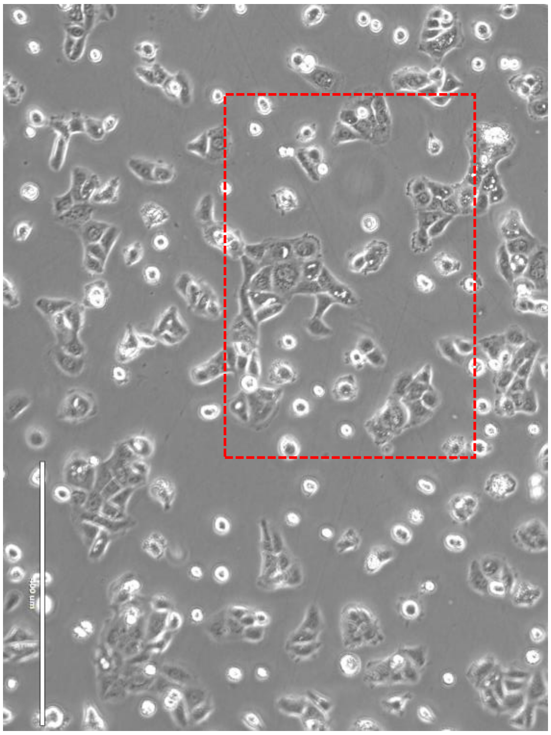

TGFβ (36 h)

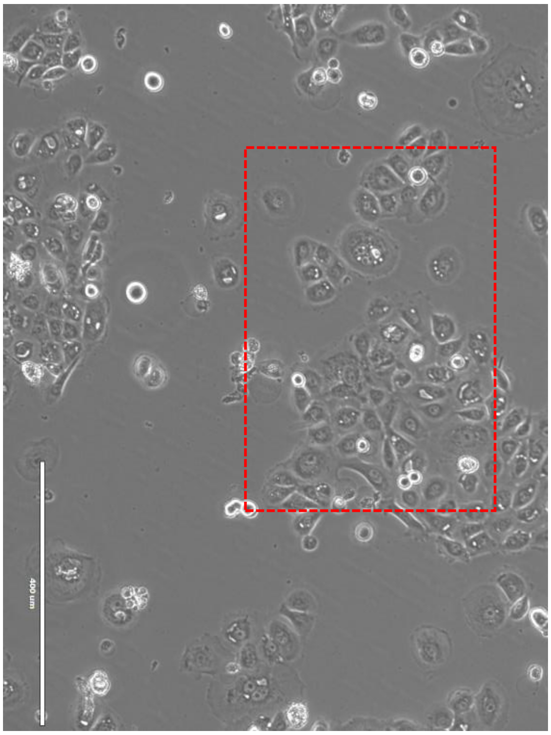

TGFβ + SB (36 h)

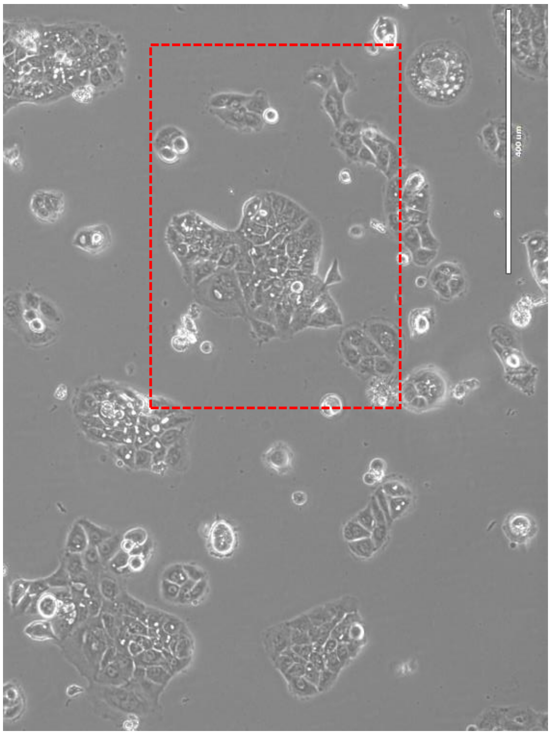

Original images for Fig. 1A
HaCaT cells treated with a range of concentrations of TGFβ1 or TGFβ2 for 24 h

Actin

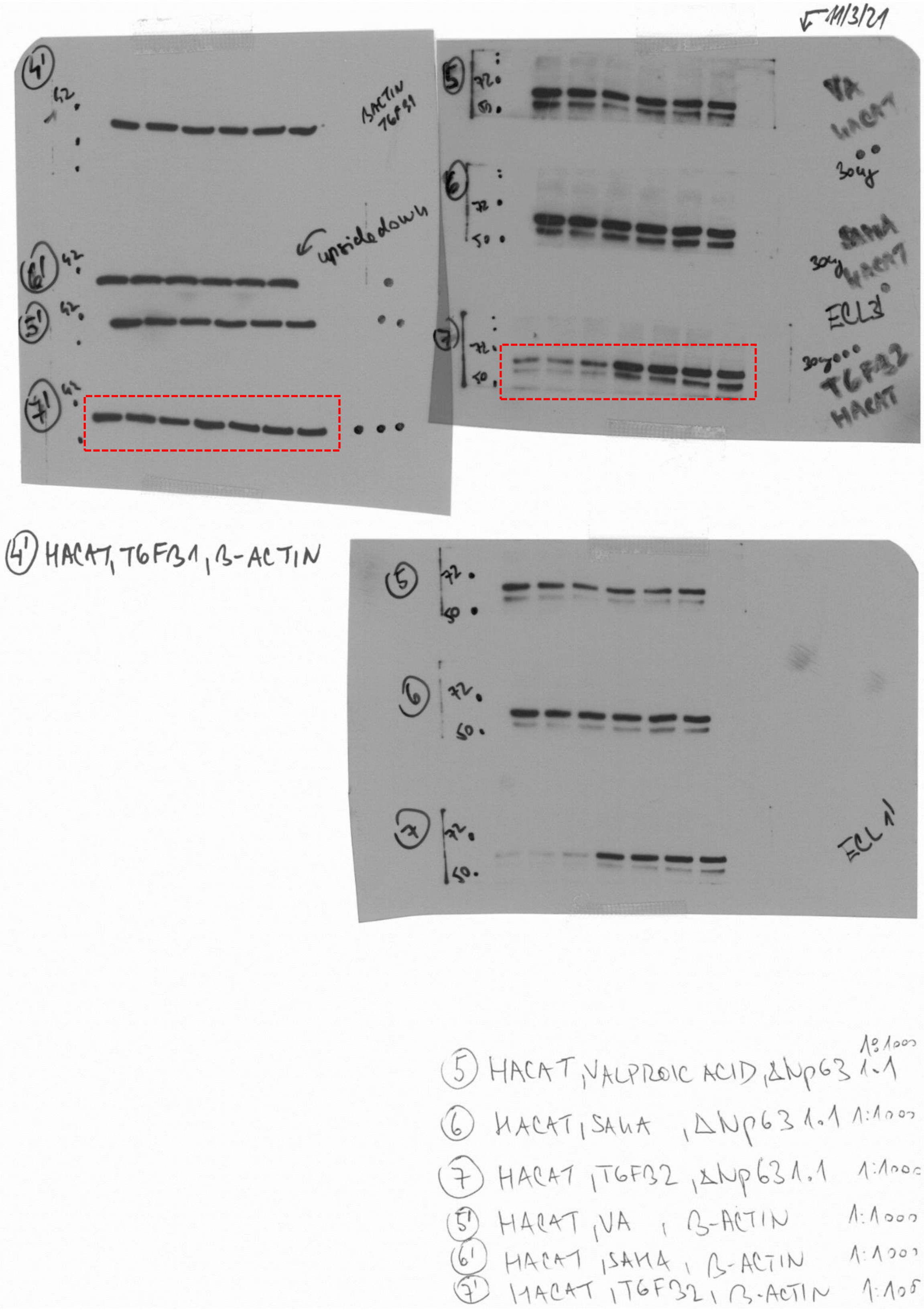

ΔNp63

Original images for Fig. 1B
FaDu cells treated with a range of concentrations of TGFβ1 or TGFβ2 for 24 h

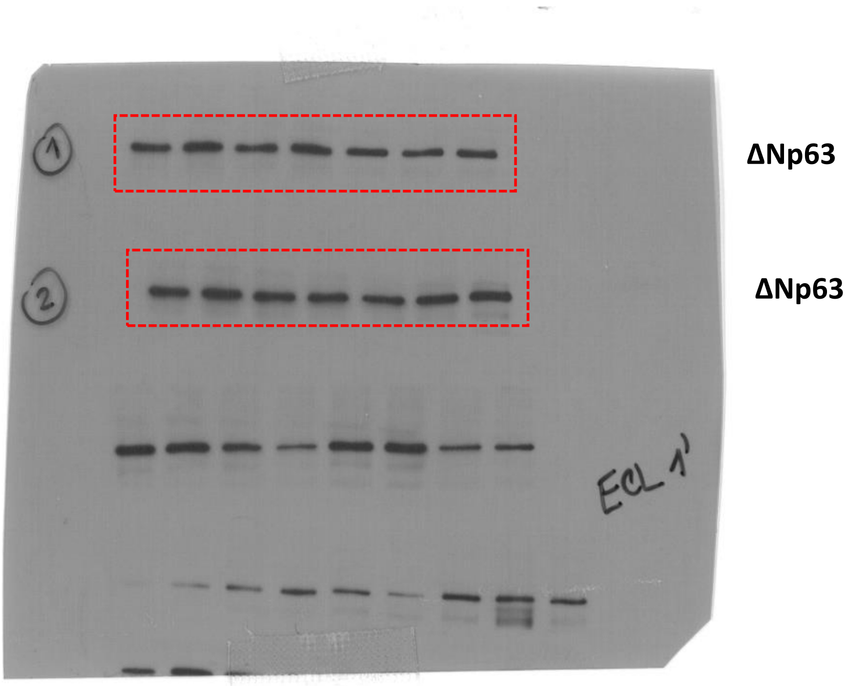

① FADu, TGFB1 (24h)
ΔNp63 1.1 1:500

② FADu, TGFB2 (24h)
ΔNp63 1.1 1:500

1', 2' FADu, TGFB 1/2
BACTIN 1:1000

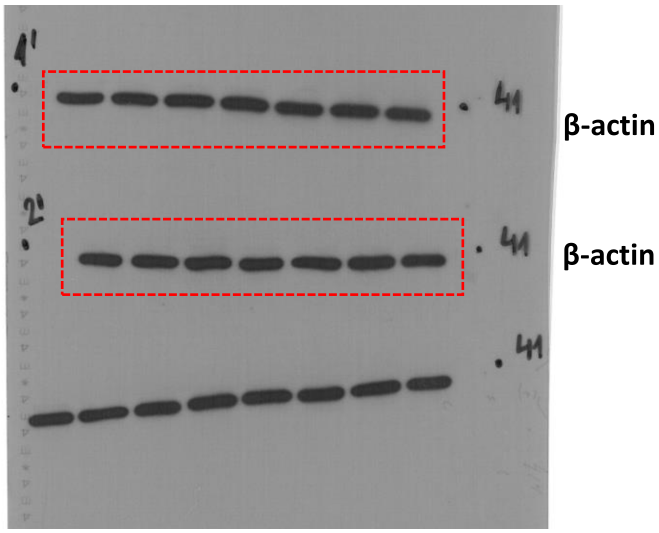

Original images for Fig. 1C

SCC-25 cells treated with a range of concentrations of TGFβ1 or TGFβ2 for 24 h

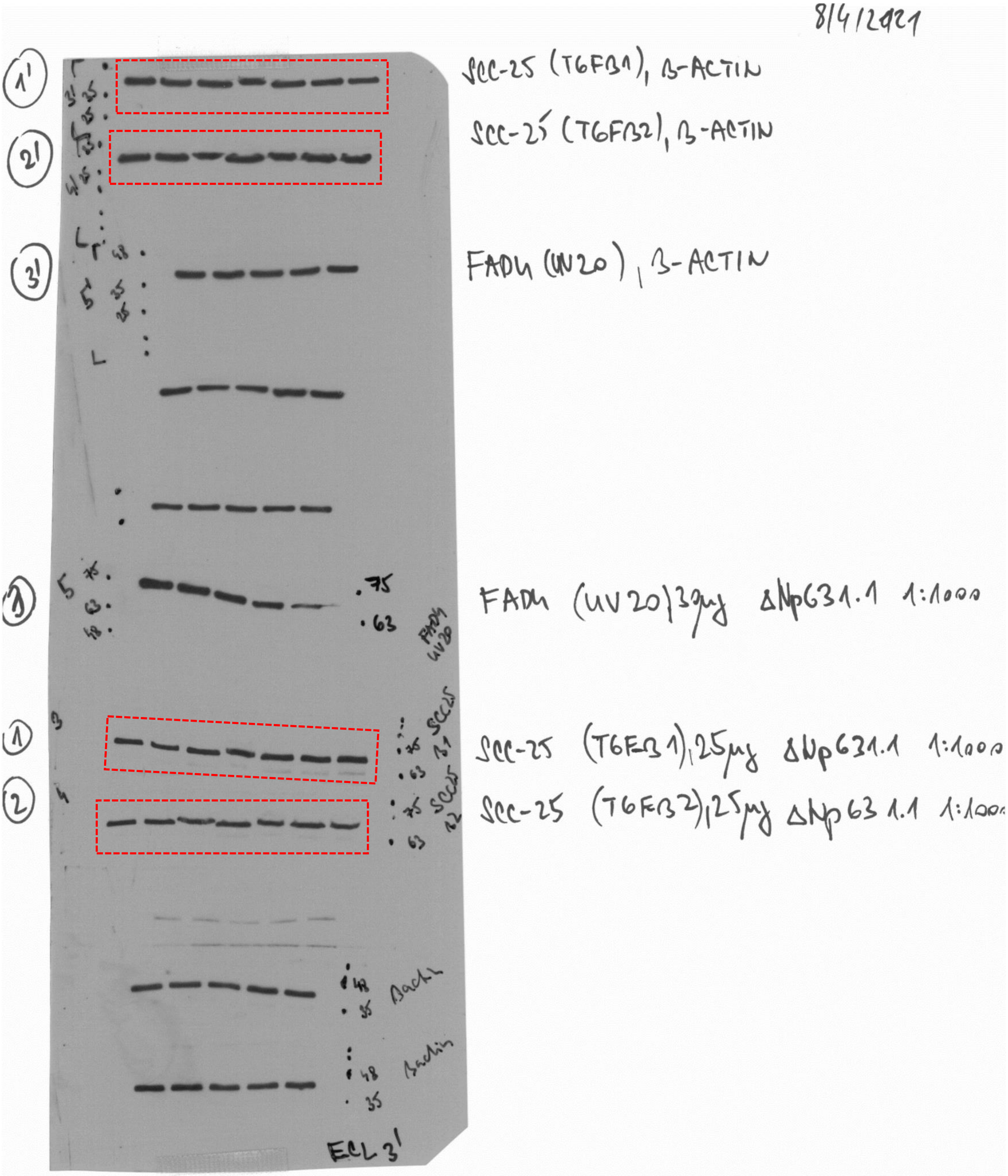

Original images for Fig. 3
HaCaT, FaDu or SCC-25 cells treated with TGFβ1 .
Westerns for ΔNp63 and β-actin

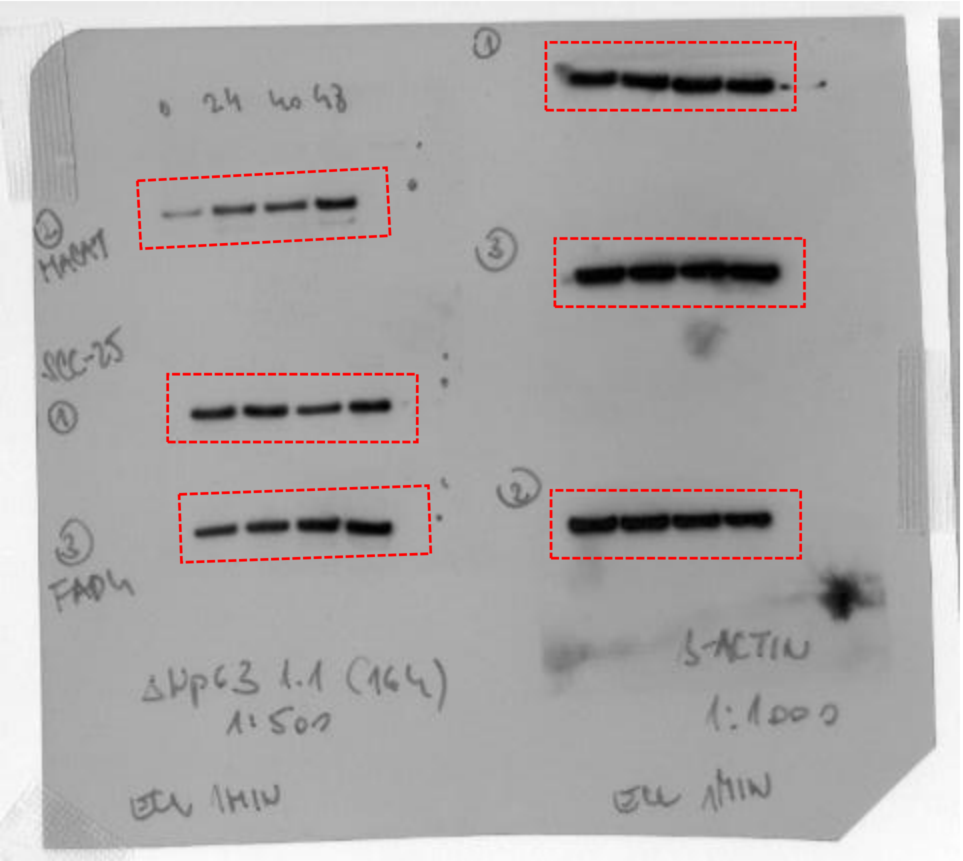

Original images for Fig. 5
Time course of HaCaT cells treated with TGFβ1 .
Westerns for ΔNp63 and β-actin

S= H1299
(control)

pSMAD3

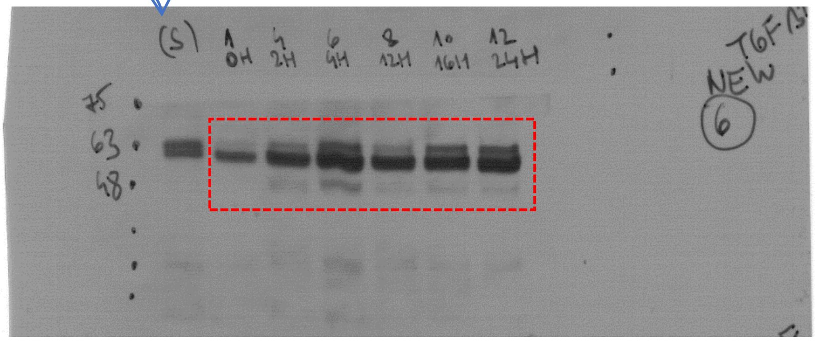

S= H1299
(control)

B-actin

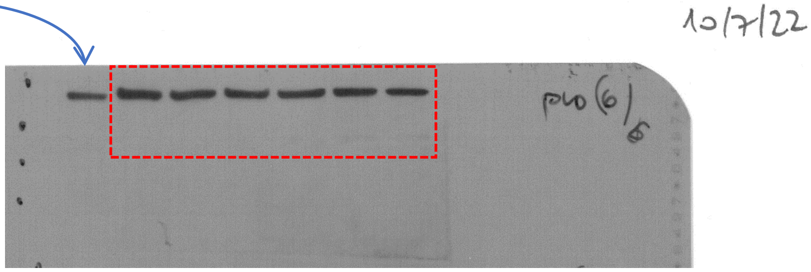

Original images for Fig. 5
Time course of FaDu and SCC-25 cells treated with TGFβ1 .
Westerns for p-SMAD3

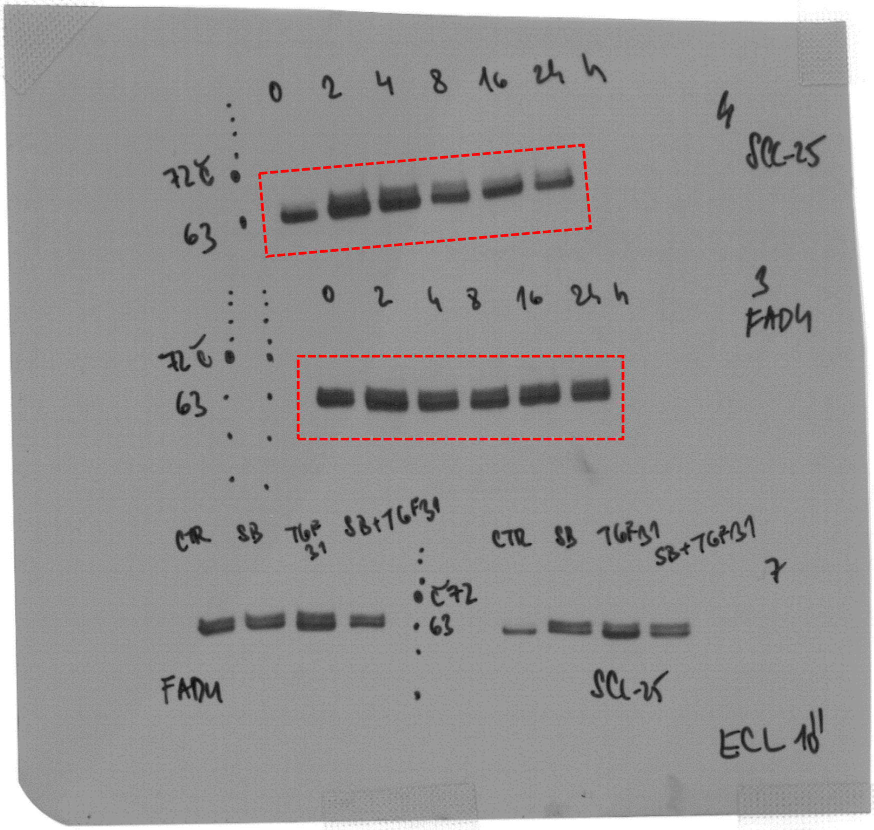

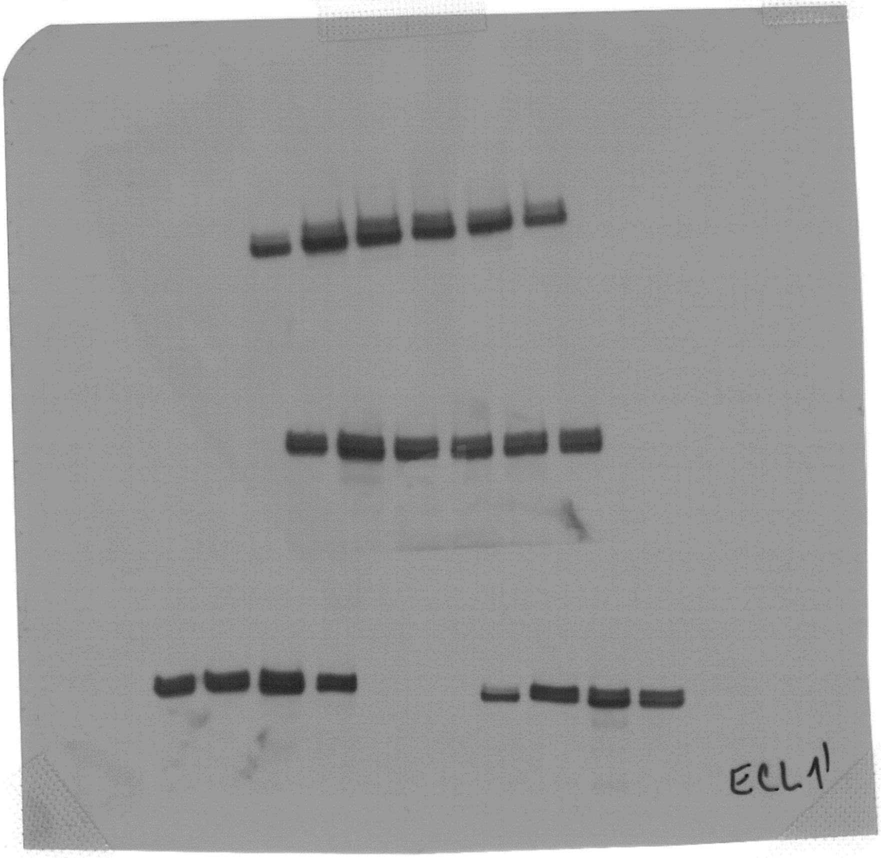

TGFβ1 20ng/ml

p-SMAD3 1:1500 (anti-rabbit) 16h
II. antibody 1:5000 (1h)

Original images for Fig. 5
Time course of FaDu and SCC-25 cells treated with TGFβ1 .
Westerns for β-actin

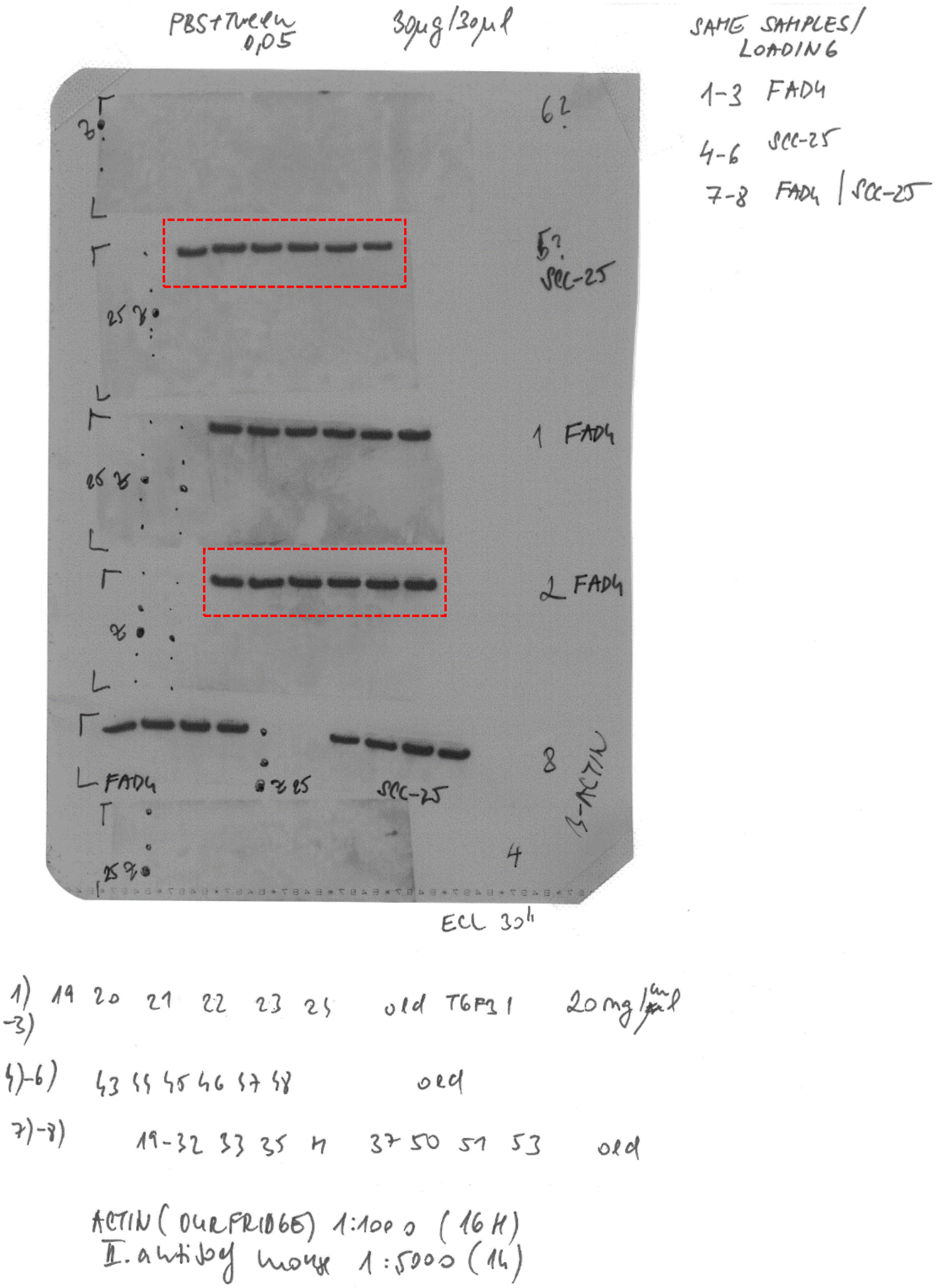

Original images for Fig. 6
HaCaT, FaDu and SCC-25 cells treated with TGFβ1 or TGFβ2, with or without SB431542.
Westerns for ΔNp63 and β-actin

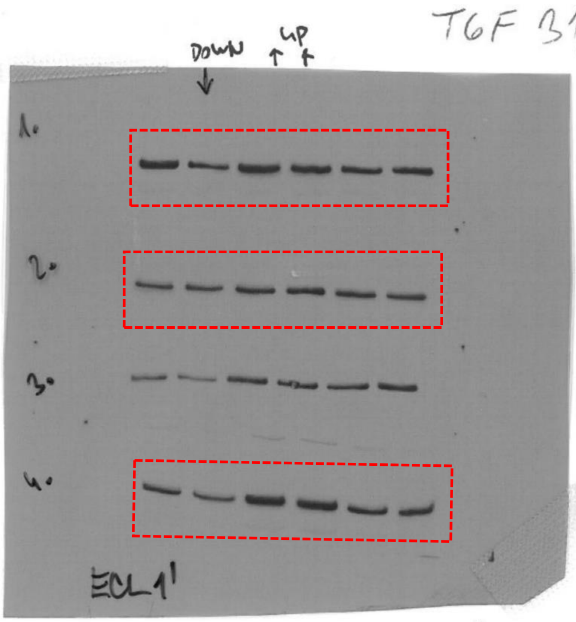

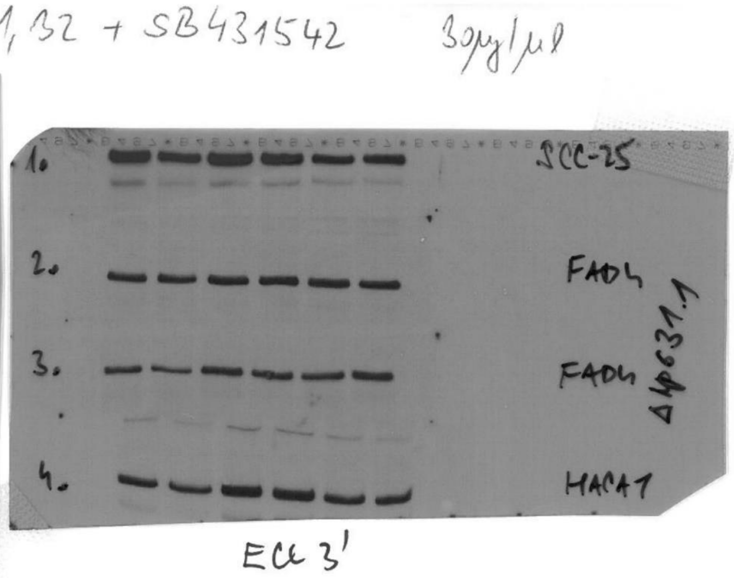

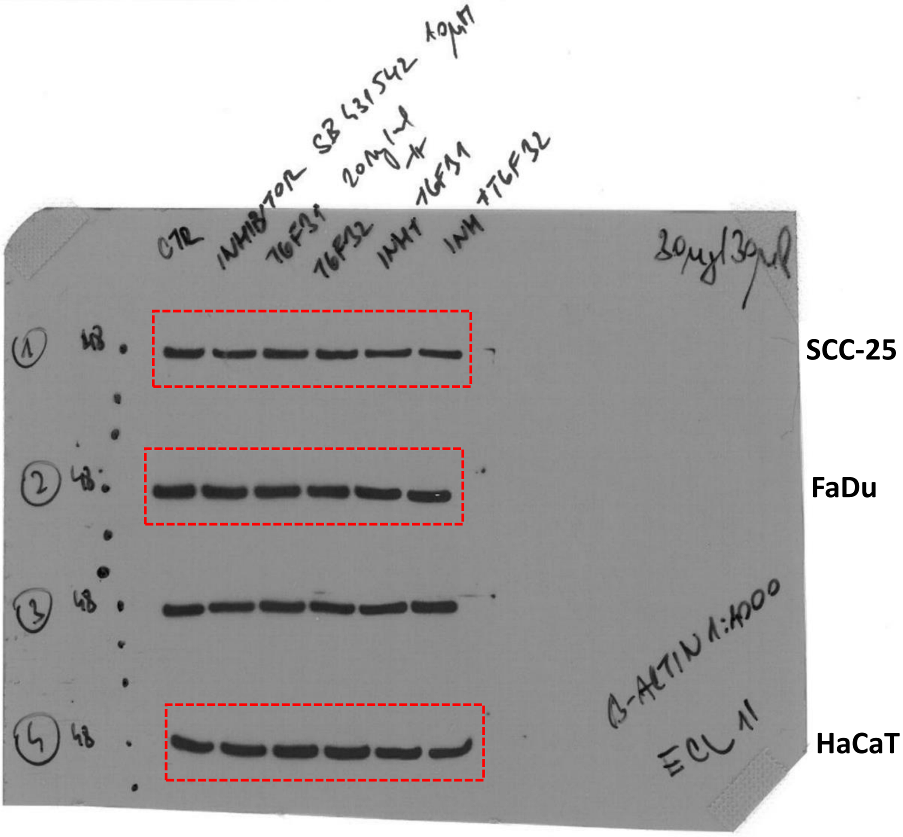

SCC-25

FaDu

HaCaT

All slots:

CTR · SB431542 10μM · TGFβ1 20ng/ml · TGFβ2 20ng/ml · SB+TGFβ1 10+20 · SB+TGFβ2 10+20

1 hour pretreatment SB431542, then coincubation 24h
