# Supplementary material for: Complex and variable regulation of ΔNp63 and TAp63 by TGFβ has implications for the dynamics of squamous cell epithelial to mesenchymal transition
Source: Sci Rep. 2024 Mar 27;14:7304. doi: 10.1038/s41598-024-57895-1 (PMC10973453; doi:10.1038/s41598-024-57895-1)
Supplement: Supplementary file 2 — Supplementary Figure 2. [file 41598_2024_57895_MOESM2_ESM.pdf]

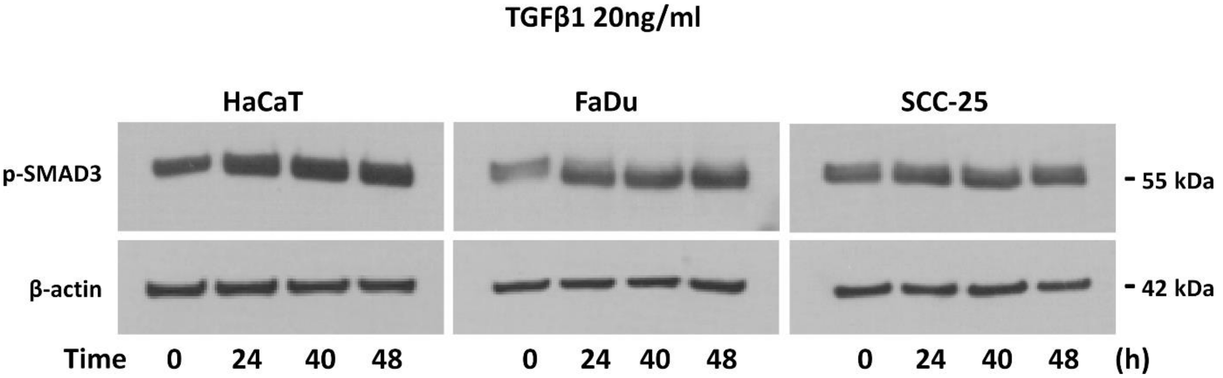

Figure S1. ***TGFβ-induced SMAD3 phosphorylation persists in HaCaT cells*****.** Representative Western blots for p-SMAD3 in HaCaT, FaDu or SCC-25 cells treated with 20 ng/ml TGFβ1 for 0, 24, 40 or 48 h.

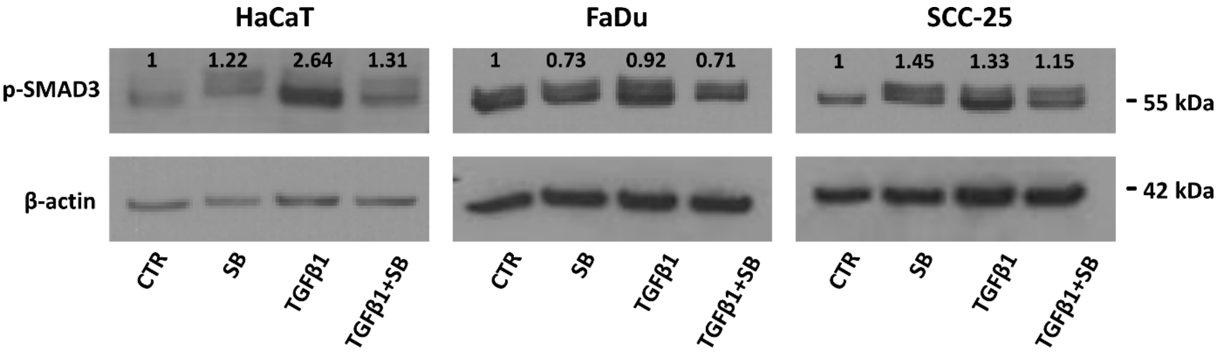

Figure S2. ***TGFβ-induced SMAD3 phosphorylation requires TGFBR*****.** Representative Western blots for p-SMAD3 in HaCaT, FaDu or SCC-25 cells. Cells were grown for 24 h in the absence of either TGFβ1 or SB435142 inhibitor (CTR); pretreated for 1 h with 10 µM SB435142 and grown for 24 h in the presence of the inhibitor (SB); grown in the presence of 20 ng/ml TGFβ1 for 24 h (TGFβ1); or pretreated for 1 h with 10 µM SB435142 and grown in the presence of both 20 ng/ml TGFβ1 and 10 µM SB435142 (TGFβ1 + SB).

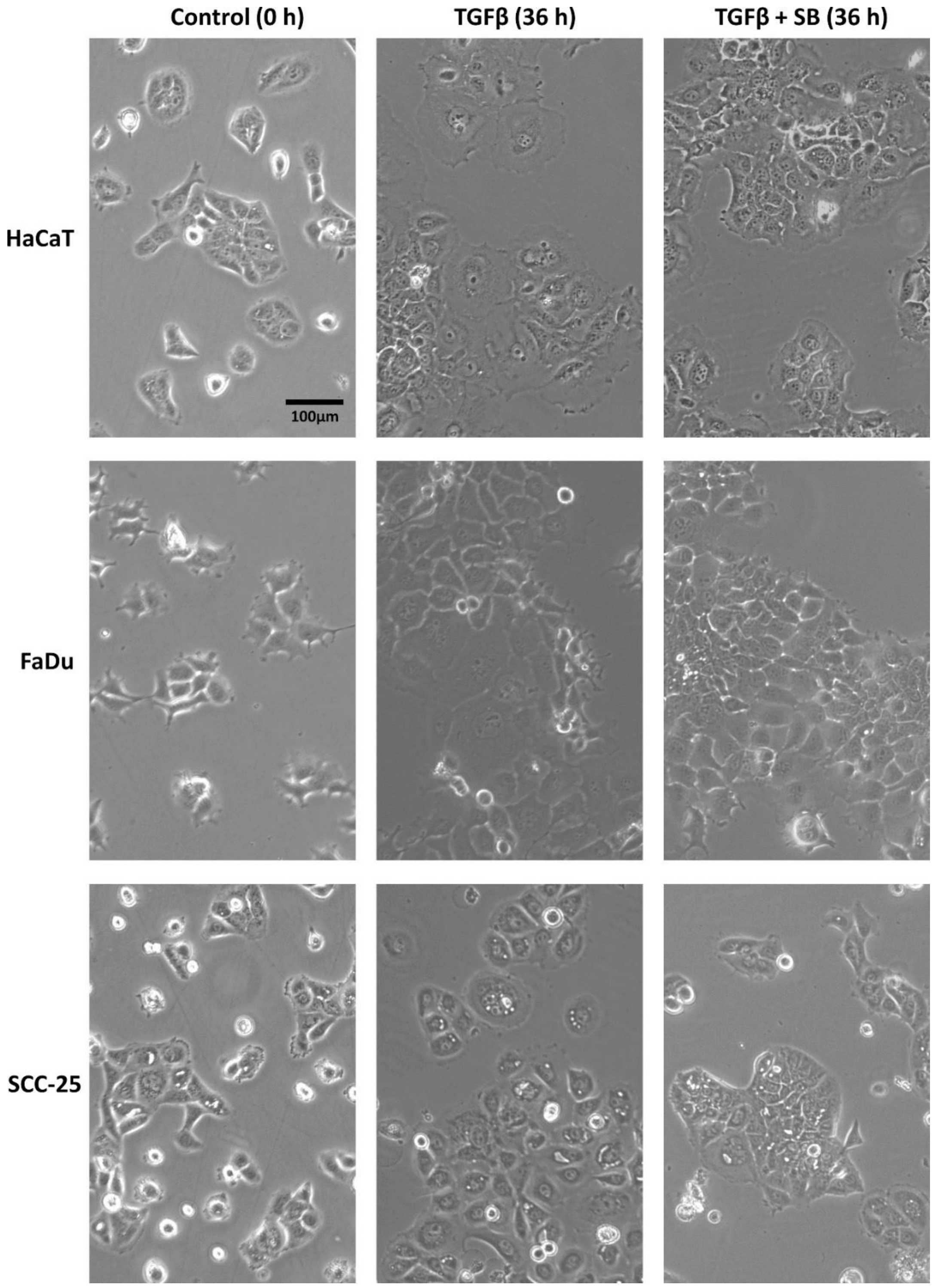

Figure S3. ***Morphological changes induced by TGFβ*****.** Cells were plated at low density and allowed to attach for 24 h before treatment (0 h) or were cultured for a further 36 h in the presence of 20 ng/ml TGFβ1, or 20 ng/ml TGFβ1 + 10 μM SB435142. Cells were photographed at the same magnification under phase contrast microscopy.
